# Cross-domain signal grammars of transcription termination

**DOI:** 10.1007/s44307-026-00134-7

**Published:** 2026-10-16

**Authors:** Shouqing Hou, Hongru Ma, Xuye Lang, Yuan Yao

**Affiliations:** 1 Department of Food Science and Engineering, School of Agriculture and Biology, Shanghai Jiao Tong University, Shanghai, China; 2 Zhejiang Key Laboratory of Intelligent Manufacturing for Functional Chemicals, ZJU-Hangzhou Global Scientific and Technological Innovation Center, Zhejiang University, Hangzhou, China

**Keywords:** Transcription termination, RNA polymerase, RNA processing, Machine learning, Terminator engineering

## Abstract

Transcription termination establishes gene boundaries, recycles RNA polymerase, and prevents interference between neighboring transcription units. Termination depends on cis-acting elements, trans-acting factors, and the susceptibility of the elongation complex to pausing, cleavage, destabilization, or release. Predicted motifs, stable RNA ends, processing sites, readthrough regions, and polymerase-release regions are nevertheless often treated as measurements of the same event, which complicates mechanistic comparison and model development. This review compares termination signals as context-dependent rule systems in which sequence determinants, RNA structure and accessibility, and processing sites are interpreted through their order and spacing, molecular readers, conditional gates, and kinetic windows. It examines bacterial intrinsic and Rho-dependent termination, archaeal U-rich and cleavage-linked routes, PAS-centered RNAPII termination, and nuclear Pol I, Pol III, and specialized RNAPII pathways, showing how shared kinetic constraints are met by divergent molecular solutions shaped by cellular organization. Matching each signal feature to the measurement that supports it clarifies cross-domain comparisons and guides transcriptome-wide analysis, prediction, and terminator engineering in defined biological systems.

**Supplementary Information:**

The online version contains supplementary material available at https://doi.org/10.1007/s44307-026-00134-7.

## Introduction

Transcription-unit boundaries depend on cis-acting termination signals carried by the DNA template or nascent RNA. Here, the term refers to locally acting sequence motifs, RNA structures, accessible RNA regions, or processing elements that influence pausing, cleavage, destabilization, or release within the same transcription unit. Yet a terminator may denote a predicted hairpin, mapped RNA 3′ end, poly(A) site, readthrough region, or polymerase-release region, preventing direct cross-system comparison (Ray-Soni et al. 2016; Porrua et al. 2016; Roberts 2019). The central question is which of these cis-acting features specify when and where RNAP, termination factors, or RNA-processing machinery act.

This comparison is difficult because the systems are organized differently: bacterial intrinsic elements are compact, Rho-dependent signals are distributed, archaeal routes can couple U-rich information to cleavage, and RNA polymerase II (RNAPII) separates polyadenylation signal (PAS) recognition, RNA processing, and release (Porrua et al. 2016; Maier and Marchfelder 2019; Wenck and Santangelo 2020). The same diversity affects experimental interpretation and computational labels. Section 2 therefore distinguishes candidate motifs, RNA ends, cleavage sites, readthrough, and measurements of polymerase release before the system-specific mechanisms are considered.

Since the cross-domain synthesis by Porrua et al. (2016), structural studies, transcriptome-scale mapping, archaeal cleavage-linked mechanisms, and computational approaches have expanded the evidence available for comparison (Porrua et al. 2016; You et al. 2023, 2024; Molodtsov et al. 2023; Stroup et al. 2025; Kosiński et al. 2025). We use “signal grammar” as an analytical framework for the system-specific rules that relate cis-acting elements to their order and spacing, molecular readers or effectors, conditional gates, kinetic competition, and measured outcome. A bacterial hairpin followed by a U-tract and an RNAPII PAS region with auxiliary upstream and downstream elements therefore represent different grammars: their components are ordered differently, decoded by different machinery, and connected to release through different spatial and kinetic relationships (Neve et al. 2017; Roberts 2019; Rodríguez-Molina et al. 2023).

The review covers bacterial intrinsic and Rho-dependent termination, archaeal U-rich and cleavage-linked routes, and eukaryotic nuclear termination, including RNAPII PAS-centered and alternative routes, Pol I, Pol III, and specialized RNAPII pathways. It first defines the comparison terms, then examines these systems, the shared kinetic constraints and cellular organization underlying their divergent solutions, the limits of evolutionary inference, and the implications for prediction, validation, and terminator design.

## Analytical concepts and evidence framework for termination signals

Cross-system comparison requires a vocabulary that separates biological signals from the assays used to infer them. This section defines the terms used throughout the review and distinguishes signal features from the measurements used to assess them. It also explains how the strength of a conclusion depends on the type of evidence available, enabling comparison of systems studied with different methods.

### Terminators, termination signals, and termination mechanisms

A terminator is a region associated with polymerase release or stable transcript-end formation; a cis-acting termination signal is a locally acting feature carried by the DNA template or nascent RNA that biases an elongation complex toward pausing, processing-associated transitions, destabilization, or release; and a termination mechanism is the broader causal process involving RNAP, trans-acting factors, RNA-processing enzymes, and kinetic or cellular context. A candidate signal or mapped transcript end does not by itself identify the release mechanism (Ray-Soni et al. 2016; Porrua et al. 2016; Wenck and Santangelo 2020).

Intrinsic bacterial termination is often associated with a local hairpin/U-tract arrangement, although its activity depends on sequence context and the elongation complex. Rho-dependent termination instead relies on a Rho utilization (*rut*) region together with RNA accessibility, translation status, RNAP pausing, and a downstream kinetic window. Archaeal U-rich regions can contribute to intrinsic-like destabilization, cleavage-linked termination, or transcript-end formation. In RNAPII systems, PAS recognition and cleavage establish RNA processing, whereas polymerase release typically occurs further downstream (Maier and Marchfelder 2019; Roberts 2019; Rodríguez-Molina et al. 2023).

### Signal features and an operational grammar

Termination signals can carry four non-exclusive kinds of information: sequence motifs (U-tracts, rut-like composition, U-rich regions, and PAS elements); RNA structures (hairpins and competing folds); RNA accessibility (factor access shaped by folding, ribosomes, and RNA-binding proteins); and processing sites (archaeal cleavage regions and eukaryotic cleavage/polyadenylation sites).

In this review, a termination signal grammar is a system-specific, context-dependent rule set with five operational dimensions: elements, comprising the four feature classes; syntax, comprising their orientation, order, spacing, and local organization; readers or effectors, such as RNAP, Rho, aCPSF1/FttA-family factors, CPA machinery, or Xrn2/Rat1; conditional gates, including RNA folding, translation status, chromatin, polymerase state, host, and cellular condition; and kinetic rules, which describe competition among signal formation, factor loading, cleavage, pausing, translocation, and elongation-complex escape. These dimensions alter the probability or position of outcomes such as pausing, cleavage, stable RNA-end formation, readthrough, a release window, or polymerase release. A list of features is therefore not by itself a grammar. The rules can involve co-requirement, constrained order, spacing dependence, context gating, alternative routes, or graded effects; these are probabilistic biological relationships rather than a rigid Boolean code.

Determinant weighting and evidence strength are treated as separate axes. Mechanistic role distinguishes features that participate in the proximal release-commitment process, recognition or coupling requirements that enable that process, and context-dependent modulators of its efficiency. Evidence level instead indicates how closely an experimental approach observes polymerase release; it does not assign universal biological importance to the feature being measured. Mechanistic priority must therefore be evaluated within each termination system and route.

**Fig. 1:**
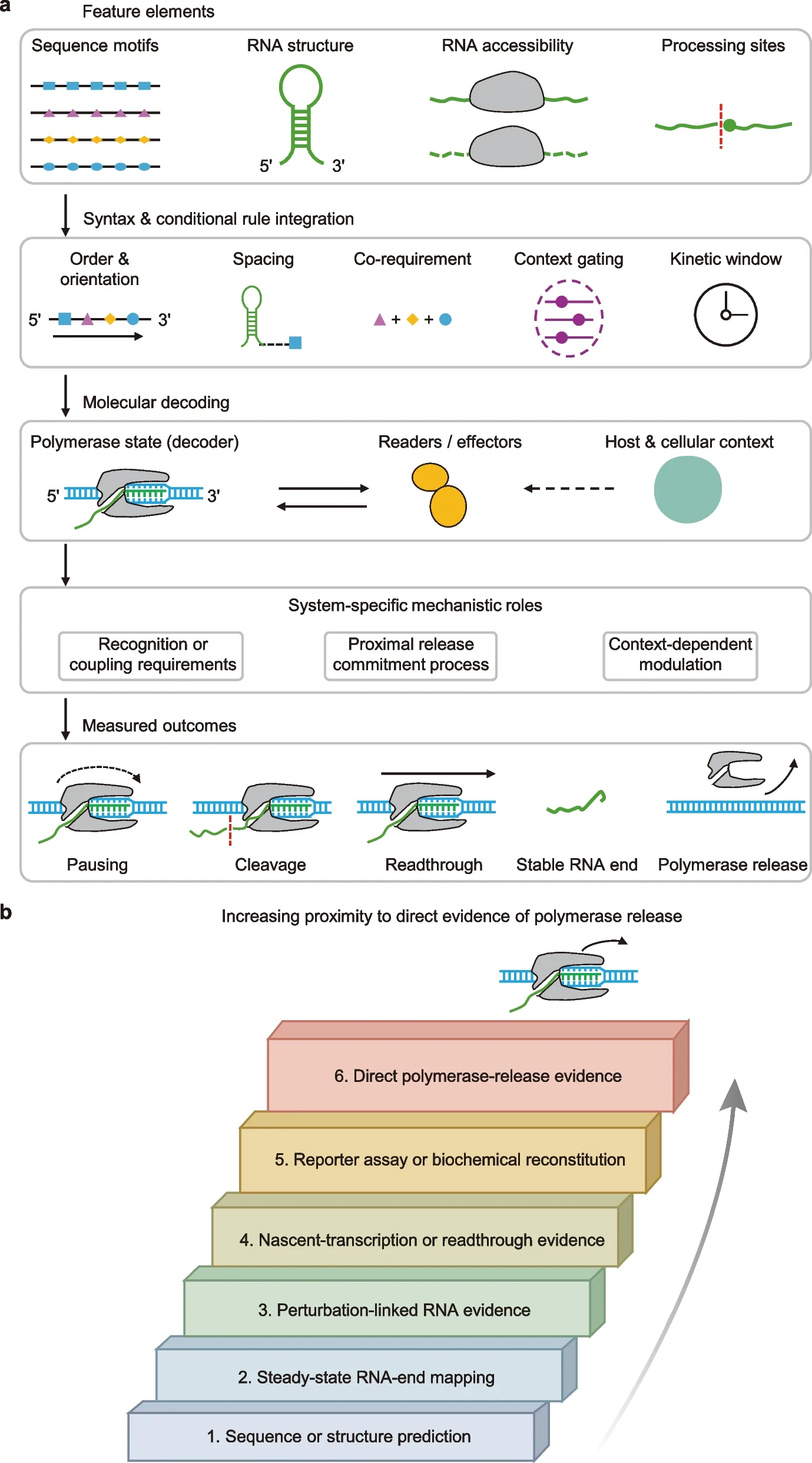
Operational termination-signal grammar and evidence-strength framework. **a** Sequence motifs, RNA structure, RNA accessibility, and processing sites form the elements of a system-specific grammar. Their orientation, order, spacing, conditional gates, and kinetic relationships determine how molecular readers or effectors connect them to pausing, cleavage, readthrough, stable RNA-end formation, or polymerase release. Recognition or coupling requirements, proximal release-commitment processes, and context-dependent modulation are shown as parallel mechanistic roles whose assignment depends on the system and route, not as a universal temporal sequence. Table 1 denotes the corresponding roles as Enabling, Proximal, and Modulators. **b** The evidence framework orders prediction, RNA-end mapping, perturbation, nascent-transcription or readthrough measurements, reporter or biochemical assays, and measurements that directly monitor polymerase release by their proximity to that endpoint. Unless otherwise indicated, DNA is shown in blue and RNA in green, and both are oriented 5′ to 3′

The same dimensions produce different rules in each system. An intrinsic bacterial hairpin must form upstream of a U-tract while the elongation complex remains susceptible to release; an accessible *rut* region must support Rho loading and translocation before a downstream complex escapes; archaeal U-rich information can enter alternative intrinsic-like or cleavage-linked routes; and RNAPII PAS recognition and cleavage precede release within a downstream window (Roberts 2019; Wenck and Santangelo 2020; Song et al. 2022; Rodríguez-Molina et al. 2023). For example, the intrinsic grammar predicts that disrupting hairpin-before-U-tract order or shortening the pause window will reduce release even when both sequence features remain present, whereas restoring productive spacing or pausing should rescue termination. Figure 1a separates recognition or coupling requirements, proximal release-commitment processes, and context-dependent modulation as parallel, system-specific roles. Table 1 applies the corresponding Enabling, Proximal, and Modulators labels to each representative system.

**Table 1:** Operational comparison of termination-signal grammars across representative transcription systems

System	Core signal and syntax	Mechanistic weighting	Evidence/limit
Bacterial intrinsic	Hairpin → U-tract during proximal pause	Proximal: hairpin completion/hybrid disruption; Enabling: pause/spacing; Modulators: folds, RNAP state, factors, host	Structure/release + reporter; RNA end ≠ release
Bacterial Rho-dependent	Accessible C-rich/G-poor *rut* → pause zone	Proximal: Rho-TEC engagement; Enabling: *rut* loading/translocation; Modulators: translation, folding, NusG, pause	Structure/single molecule/biochemistry; readthrough ≠ release site
Archaeal U-rich/cleavage-linked	U/T-rich tract → intrinsic-like or cleavage-linked route	Proximal: hybrid disruption/cleavage-linked route; Enabling: U-rich recognition/nuclease coupling; Modulators: lineage, factors, processing	Perturbation/reconstitution/structure; RNA end or cleavage ≠ route
RNAPII PAS-centered	PAS + auxiliary elements → cleavage → release window	Proximal: post-cleavage slowing + Xrn2/Rat1; Enabling: CPA cleavage; Modulators: PAS strength, elongation, chromatin	Nascent/factor assays; poly(A) site ≠ release
Pol I	Factor-bound rDNA terminator; T-rich region/hairpin vary	Proximal: pause + hairpin/factor destabilization; Enabling: factor binding; Modulators: species, template, surveillance	Release/occupancy assays; RNA ends or models ≠ universal route
Pol III	Poly(dT)/oligo(U); nearby hairpin strengthens weak sites	Proximal: pause + hybrid destabilization; Enabling: Pol III recognition; Modulators: tract, hairpin, Sen1	Cryo-EM/in vitro; final transitions partly modeled
Specialized RNAPII	NNS/Sen1, Integrator-snRNA, or histone PAS/HDE	Proximal: Sen1 or processing-linked route; Enabling: recruitment/processing; Modulators: transcript class, CTD, promoter/context	Recruitment/processing/readthrough ≠ Pol II-DNA release

The table compares core signal and syntax, mechanistic weighting, and evidence limits for bacterial intrinsic, bacterial Rho-dependent, archaeal U-rich or cleavage-linked, RNAPII PAS-centered, Pol I, Pol III, and specialized RNAPII termination. The Proximal category identifies the process closest to release commitment, Enabling covers recognition or coupling requirements, and Modulators include context-dependent influences. These roles are assigned within each system and remain distinct from evidence strength

### Experimental measurements and the evidence hierarchy

A predicted motif or structure is a candidate signal. An RNA 3′ end is a mapped transcript boundary that may result from primary termination, cleavage, processing, protection, or decay. A cleavage or poly(A) site identifies an RNA-processing event but does not by itself locate RNAPII release. Readthrough measures transcriptional or RNA accumulation beyond a reference region, whereas a termination window describes a region over which polymerase-associated signal declines. Direct polymerase release is the narrowest mechanistic endpoint, but it is measured less often at genome scale.

To make these inference limits explicit, this review uses a six-level evidence framework that orders established experimental approaches by their proximity to direct observation of polymerase release. Level 1, sequence or structure prediction, identifies candidate features but does not establish function. Level 2, steady-state RNA-end mapping, adds positional evidence while remaining sensitive to processing and decay. Level 3, perturbation-linked RNA evidence, connects an end or readthrough phenotype to a factor or condition. Level 4, nascent-transcription or readthrough evidence, more directly localizes changes in polymerase progression. Level 5, reporter assays or biochemical reconstitution, tests function in defined experimental contexts. Level 6, direct polymerase-release evidence, directly addresses the release step under the measured conditions. These approaches address different parts of the termination process and can be combined when they provide concordant evidence. Conclusions should nevertheless match the measurement and limitations of the methods used (Fig. 1b; Table 2).

**Table 2:** Experimental methods, measurements, and limits of inference in transcription-termination studies

Assay or method	What it supports	What it does not establish
Sequence or structure prediction	Candidate prioritization with biological context	Mechanism, release, or portability from prediction alone
Term-seq and related 3’-end sequencing	Termination-associated end when sequence and orthogonal evidence converge	Primary termination or release without processing/decay controls
TERMITe or PIPETS analysis	Intrinsic interpretation with paired RNA-seq, sequence, or perturbation	Mechanism from every detected peak
Cleavage/poly(A)-site profiling	Processing change when supported by downstream nascent or release data	RNAPII release location or mechanism from cleavage maps alone
Long-read or direct RNA sequencing	Isoforms, RNA-product lengths, readthrough with controls	Release position or mechanism from RNA products alone
Factor depletion or perturbation with RNA-end measurements	Pathway involvement with binding, rescue, nascent, or reconstitution evidence	Direct signal recognition or a unique causal route
Nascent-transcription profiling or readthrough analysis	Release when linked to cis-element perturbation, factor dependence, or direct-release assays	Single-nucleotide release coordinate or complete mechanism
Total RNA-seq readthrough or DoG analysis	Altered progression with nascent, kinetic, processing, and stability controls	Immediate failure or release location from accumulated RNA alone
Reporter readthrough assay	Function in the tested construct and host	Universal portability, native behavior, or precise release route
Biochemical reconstitution or in vitro termination	Mechanism in the reconstituted system	Genome-wide prevalence or universal cellular behavior
Cryo-EM or structural analysis	A pathway model with complementary kinetic or biochemical evidence	An obligatory pathway or universal mechanism
Single-molecule transcription or RNA-product assay	Trajectories, kinetics, or product-length distributions	Release only if dissociation observed; not genome-wide
Direct polymerase-release assay	Release on the tested template and system	Universal mechanism, prevalence, or portability

Comparison of prediction, mapping, perturbation, nascent-transcription, reporter, biochemical, structural, and direct-release approaches. For each method, the table indicates the measurement it provides, the conclusion supported when combined with relevant evidence, and claims it cannot establish alone (Fig. 1b)

## Bacterial intrinsic terminators (hairpin + U-tract)

Bacterial intrinsic terminators use RNA-hairpin formation and a U-rich RNA–DNA hybrid to destabilize a paused elongation complex. Operationally, the inverted repeat must precede the U-tract and fold while pausing preserves a release-susceptible complex. Timely hairpin folding and hybrid disruption constitute the proximal release-commitment process, whereas terminator spacing, competing folds, RNAP conformation, accessory factors, and host context gate the probability and efficiency of release. Structural intermediates and purified-system release assays provide the closest mechanistic support for this assignment; reporter and RNA-end measurements establish function or position under their respective conditions (Bellecourt et al. 2019; You et al. 2023).

### Canonical hairpin/U-tract mechanism

Canonical intrinsic termination begins when a GC-rich inverted repeat folds into an RNA hairpin upstream of a U-rich tract. The hairpin remodels the RNAP exit channel, while the weak rU-dA-rich hybrid favors transcript release after terminator pausing (Ray-Soni et al. 2016; Roberts 2019; You et al. 2023). Timely folding, U-tract quality, and local sequence features that alter competing folds or terminator spacing determine whether this state proceeds to release.

**Fig. 2:**
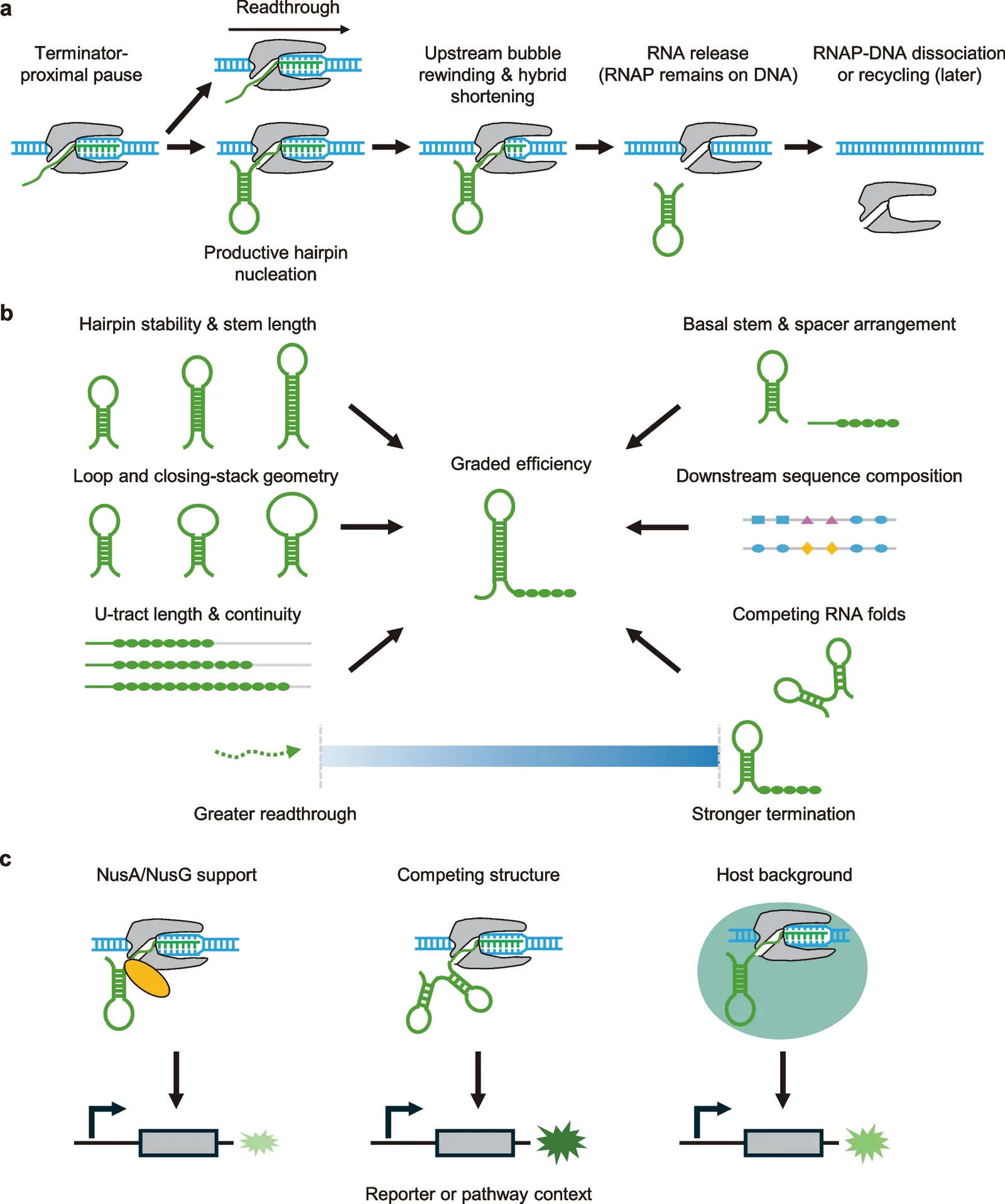
Compact bacterial intrinsic termination grammar. **a** This simplified mechanistic synthesis begins with a terminator-proximal pause and productive hairpin nucleation after the relevant RNA has emerged into the RNA exit channel. Failure to complete this pathway before RNAP escapes the kinetic window produces readthrough. Along the termination branch, short paired blue arrows indicate local DNA re-pairing from the upstream edge of the transcription bubble; the resulting hybrid shortening or destabilization facilitates hairpin completion and RNA release. RNA release is shown separately from subsequent RNAP-DNA dissociation or recycling. Black arrows indicate mechanistic progression or alternative outcomes. **b** Termination efficiency varies along a continuum shaped by hairpin stability and stem length, loop and closing-stack geometry, U-tract length and continuity, basal-stem and spacer arrangement, downstream sequence composition, and competing RNA folds. **c** Accessory-factor support, competing structures, host background, and reporter or pathway context can alter the activity of the same core module. Efficiency and cross-host portability therefore require direct testing in the intended biological and assay context

Cryo-EM structures of *Escherichia coli* intrinsic termination complexes capture a sequence-induced pause, hairpin nucleation after the relevant nascent RNA has emerged into the RNA exit channel, and later intermediates associated with transcript release (You et al. 2023). At the terminator-proximal pause, the RNA–DNA hybrid is half-translocated and the hairpin is incomplete. Local re-pairing of template and non-template DNA from the upstream edge of the transcription bubble can shorten or destabilize the upstream end of the hybrid, facilitating further hairpin completion and RNA release. If productive hairpin formation and the associated nucleic-acid rearrangements do not occur before RNAP escapes this kinetic window, transcription instead continues as readthrough. RNA release can precede complete RNAP-DNA dissociation, consistent with biochemical evidence that RNA and DNA release are separable steps (Bellecourt et al. 2019). Figure 2a presents this sequence as a simplified synthesis of structural and functional evidence rather than one obligatory frame-by-frame trajectory.

### Quantitative strength determinants and synthetic libraries

Quantitative sequence-function studies show why intrinsic termination is graded rather than binary. A standardized *E. coli* reporter measured 61 natural and synthetic terminators across an approximately 800-fold expression range; a four-feature model explained efficiency for 31 curated canonical terminators (*r* = 0.9; cross-validated *r* = 0.85) using U-tail score, normalized hairpin stability, closing-stack identity, and downstream DNA composition (Cambray et al. 2013). Extended, weak, or kinetically constrained terminators remained poorly predicted, implicating proximal sequence context and RNA folding beyond these descriptors.

Larger libraries and FlowSeq-style experiments have expanded this picture. Sequence-diverse synthetic terminator libraries measured by qTerm-Seq identified hundreds of strong *E. coli* intrinsic terminators, many with termination efficiencies above 90%, and linked high efficiency to features such as strong hairpin pairing, favorable basal stem composition, and an extended poly(U) tract (Hudson and Wieden 2019). FlowSeq analysis of terminator mutant libraries similarly associated stronger termination with lower hairpin free energy, longer stem length, compact loop architecture, and a more perfect U-tract, while also emphasizing that readthrough is common even when strong terminators are used in reporter contexts (Zhai et al. 2022). Together, these studies show that intrinsic termination is generally strengthened by a stable hairpin and an extended, uninterrupted U-tract. The strength of this effect varies with proximal RNA structure, reporter design, host background, and whether the sequences are natural or synthetic. Termination efficiency therefore spans a continuum rather than a binary classification (Fig. 2b).

### Host context, accessory factors, and cross-host portability

Cross-host studies show that reduced hairpin/U-tract scaffolds can be engineered and tested in *B. subtilis*, *Corynebacterium glutamicum*, and *E. coli* (Cui et al. 2021). Proteobacteria-focused terminator-trap screens identified compact fragments active across tested hosts, although many lacked canonical hairpins (Amarelle et al. 2019). In *Halomonas bluephagenesis*, native and designed stem-loop/poly(U) elements tuned reporter expression, phaC expression, and polyhydroxybutyrate (PHB) accumulation (Xu et al. 2022). These examples show that performance, cross-host portability, and mechanism must be established in the intended host and assay. Additional intrinsic-terminator examples are summarized in Table S1.

Protein factors further blur the old distinction between “intrinsic” and “factor-independent.” In *Bacillus subtilis*, genome-wide 3′-end mapping after NusA depletion showed that NusA stimulates many weak or non-canonical intrinsic terminators, especially those with weak hairpins or distal U-tract interruptions (Mondal et al. 2016). NusA-dependent terminators were described as pseudo-intrinsic because their core signal resembles an intrinsic terminator but efficient termination in vivo depends strongly on an elongation factor. NusG adds another *B. subtilis*-specific layer: Term-seq across WT, NusA-depleted, nusG-deleted, and double-perturbation backgrounds showed that, in this system, NusG acts alone and cooperatively with NusA at a large fraction of intrinsic terminators, with the strongest dependence observed for suboptimal hairpins and sequence-specific pausing contexts (Mandell et al. 2021). Accessory factors are not simply external decorations on a finished signal. Instead, in at least some bacterial lineages, factors can extend the functional range of imperfect hairpin/U-tract modules by slowing elongation, stabilizing hairpin formation, or positioning the complex for release. Together with competing RNA folds, host background, and reporter or pathway context, these factor effects are experimentally testable determinants of efficiency and cross-host portability (Fig. 2c).

### Transcript-end mapping, internal termination, and context-dependent regulation

Assigning an intrinsic terminator from genome-wide data requires convergent evidence: a reproducible RNA end, a compatible hairpin/U-tract architecture, and, where possible, a targeted perturbation. TERMITe combines replicated Term-seq ends, hairpin/U-tract annotation, and optional readthrough estimates; Rend-seq with structure filters and targeted perturbations can strengthen assignments, while TRS and PIPETS extend candidate calls across standard RNA-seq and genomic contexts (Lalanne et al. 2018; Bar et al. 2023; Furumo and Meyer 2024; Kosiński et al. 2025). These approaches identify candidate sites, but assigning a specific termination mechanism still requires perturbation or functional evidence.

INTERPIN extends candidate comparisons across bacterial lineages by cataloguing roughly 25 million predicted intrinsic-termination hairpins in 12,745 genomes across ten phyla (Gupta et al. 2023). It supports hypotheses about alternative downstream sites and genome composition, but remains prediction-based; partial overlap with *B. subtilis* and *E. coli* in vivo sets cannot resolve expression, processing, or alternative-hairpin effects.

Transcript-boundary mapping and full-length profiling show that readthrough can remodel apparent operon structure and reveal bidirectional or alternative termination arrangements (Yan et al. 2018; Ju et al. 2019). Regulatory leaders add condition-dependent premature termination through antibiotic-responsive and riboswitch-like readthrough, while short stop-codon-to-terminator distances have been associated with weak transcription-translation coupling (Dar et al. 2016b; Johnson et al. 2020). *E. coli* and *Borrelia* 3′-end maps identify UTR-derived and ORF-internal RNAs consistent with premature termination or processing, and terminator libraries show that strength rankings require host-specific testing (Chen et al. 2013; Adams et al. 2021; Gale et al. 2021; Petroni et al. 2023).

In vitro RNAP perturbations further show that clamp movement can aid DNA dissociation without being strictly required for RNA release, whereas trigger-loop dynamics can stimulate termination without requiring the hairpin to perform all mechanical work (Bellecourt et al. 2019). Thus, the compact intrinsic system relies on timely hairpin folding and a U-rich hybrid to generate a vulnerable, release-prone elongation complex; its efficiency and cross-host portability also depend on local sequence, competing folds, RNAP conformation, accessory factors, and host context. Genome-wide maps and databases extend this framework across species.

## Bacterial Rho-dependent termination signals (*rut* + termination zone)

Rho-dependent termination requires accessible nascent RNA, Rho loading and ATP-dependent translocation, and engagement with a susceptible paused elongation complex before RNAP escapes. Operationally, an accessible *rut* region must permit productive Rho loading and translocation before a downstream paused complex escapes. Rho engagement with that complex is most proximal to release; translation status, ribosome shielding, RNA folding, pause lifetime, accessory factors, and growth context act as conditional gates. Structural, single-molecule, and biochemical studies provide the closest mechanistic evidence, while perturbation and readthrough measurements localize Rho-dependent effects across a distributed termination zone (Song et al. 2022; Molodtsov et al. 2023).

### *rut*-like RNA accessibility and translation coupling

**Fig. 3:**
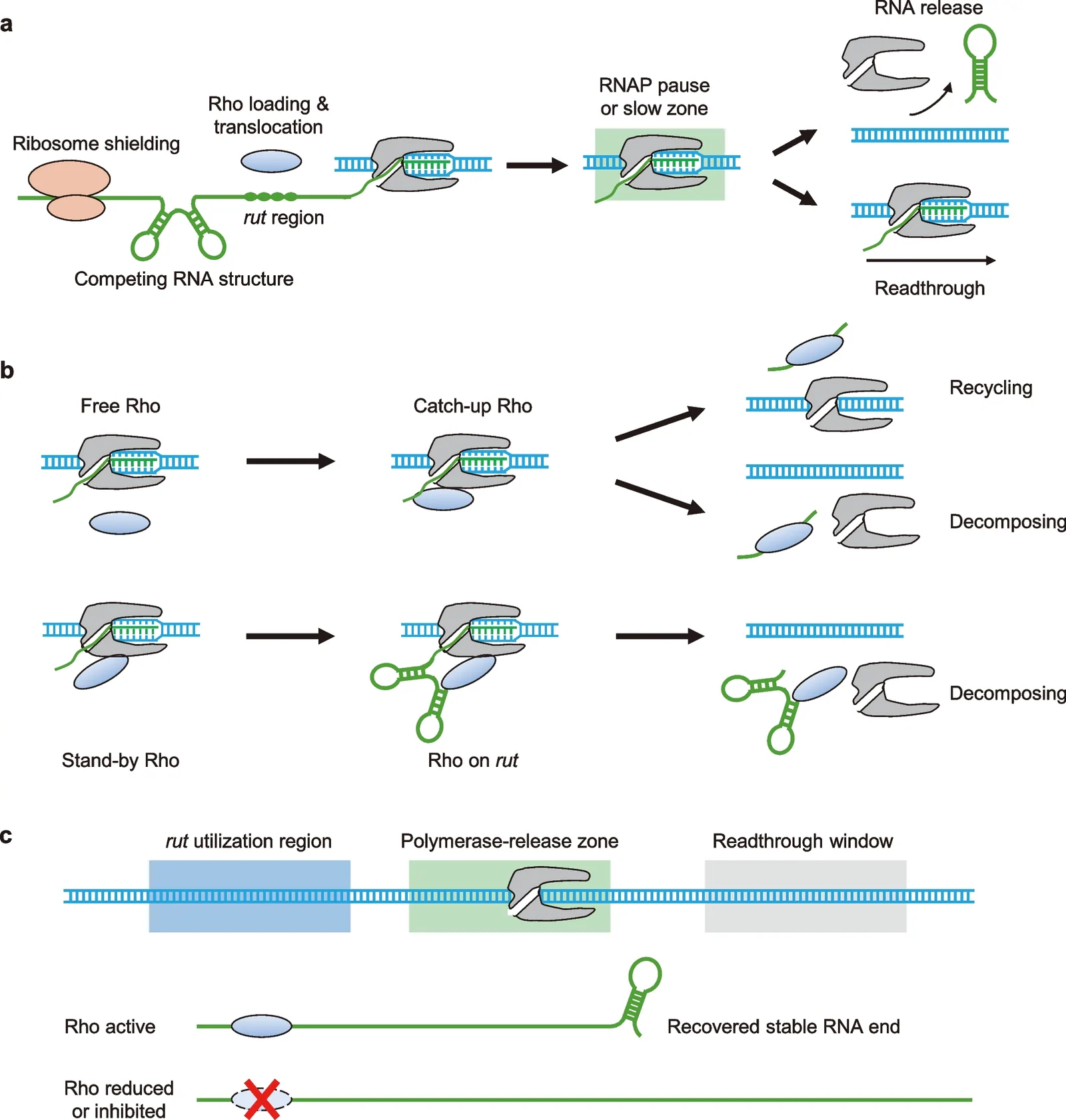
Distributed signal grammar of bacterial Rho-dependent termination. **a** Productive termination requires an exposed, often C-rich/G-poor *rut*-like RNA region, Rho loading and translocation, limited ribosome shielding, and a downstream RNAP pause or slow zone. **b** Catch-up and stand-by/pre-associated routes differ in loading position, pause duration, NusG-mediated bridging, RNA context, and recycling or decomposing outcomes. **c** The *rut* utilization region, polymerase-release region, recovered RNA end, and downstream readthrough window occupy distinct positions

Rho-dependent termination begins when the hexameric RNA-dependent ATPase Rho loads onto an exposed *rut* region of nascent RNA and catches a susceptible elongation complex. Productive termination depends on *rut* accessibility, limited ribosome shielding, RNA secondary structure, downstream polymerase pausing, and the time available before RNAP escapes (Ray-Soni et al. 2016; Kriner and Groisman 2017; Chhakchhuak and Sen 2022). Rho thus acts through an accessible RNA route and a kinetic window rather than through a compact stop element (Fig. 3a).

The best-established *rut* features are pyrimidine-rich, C-rich and G-poor RNA, often described as unstructured C > G “bubbles” long enough to engage Rho’s primary binding surface. Rho can load at a *rut* site and translocate 5′ to 3′ toward a paused RNAP, or associate earlier with RNAP and scan nascent RNA from a pre-termination complex. Both routes require productive RNA engagement, Rho activation, and an elongation complex that remains vulnerable long enough for termination to occur (Song et al. 2022; Molodtsov et al. 2023). Figure 3b contrasts catch-up with stand-by and pre-associated Rho-dependent termination routes. Structural work on *E. coli* Rho-NusG-TEC complexes visualized a catalytically competent closed Rho ring, RNA threaded through its central channel, and NusG bridging Rho to RNAP, consistent with a pre-termination complex poised for ATP-dependent 5′-to-3′ translocation toward the TEC (Molodtsov et al. 2023).

In the *Salmonella* corA leader, mutually exclusive RNA secondary structures regulate *rut* accessibility, while the same leader modulates RNAP pausing more than 100 nt downstream and uses an additional stem-loop to alter the termination position (Kriner and Groisman 2017). Thus, *rut* accessibility, downstream RNA structure, and RNAP pausing jointly shape the termination outcome. Consistent with this view, *E. coli* terminators span a wide efficiency range: weak or moderate sites correlate better with C > G-bubble descriptors and show stronger NusG dependence than strong sites (Chhakchhuak and Sen 2022).

Translation is another major determinant of Rho signal availability. Because ribosomes can shield nascent RNA, Rho often acts where translation is absent, delayed, stalled or uncoupled from transcription. EF-P-dependent relief of ribosome stalling at polyproline motifs helps maintain transcription-translation coupling and reduces premature Rho-dependent termination, showing how a translation defect can expose nascent RNA to Rho (Elgamal et al. 2016). Riboswitch and leader examples reinforce this logic: in the thiM leader, translational control and Rho-dependent termination are linked through a C-rich *rut* region and downstream RNA structures, so that translation state influences Rho access and termination outcome (Bastet et al. 2017). *Mycobacterium tuberculosis* adds a broader but species-specific version of this theme. Term-seq and Rho-depletion studies report widespread premature termination and an association between translated upstream ORFs, Rho activity, and conditional termination, but the relationship between uORF translation and Rho-dependent termination should be framed as locus-specific association unless direct perturbation evidence is available (D’Halluin et al. 2023).

Transcription-translation coupling regulates Rho-dependent termination primarily by controlling access to nascent RNA. Actively translating ribosomes can mask *rut* regions, whereas untranslated RNA, delayed translation, or ribosome stalling can expose Rho-loading sites. Whether this exposure produces termination further depends on RNA structure, Rho engagement, downstream RNAP pausing, and the duration of the kinetic window (Fig. 3a).

### Genome-wide Rho action, termination-zone inference, and prediction

Genome-wide mapping identifies Rho as a broad genome-management factor in *E. coli*, where it directs intragenic and stable-RNA termination and suppresses pervasive antisense transcription with NusG (Peters et al. 2009, 2012). Conditional depletion and complementary occupancy, readthrough, and in vitro studies indicate unusually strong reliance on Rho in *Mycobacterium tuberculosis* (Botella et al. 2017; Ahmad et al. 2023). Its balance with intrinsic termination nevertheless remains species- and assay-specific.

High-resolution *E. coli* mapping revealed a Rho-specific separation between the polymerase-release region and the recovered transcript end: locally precise steady-state 3′ ends often lie near protective stem-loops lacking the U-rich tract expected for intrinsic termination (Dar and Sorek 2018). Evidence that these structures protect processed transcripts from 3′-to-5′ exonucleases indicates that the mapped endpoint can be downstream of, and mechanistically distinct from, Rho utilization and release. The *rut* utilization region, broad polymerase-release region, recovered stable RNA end, and downstream readthrough window occupy distinct spatial positions (Fig. 3c). This region-versus-end distinction motivates synthetic small RNA stabilization of Rho-terminated fragments and H-SELEX mapping of *rut*-like sequences, which seek more direct localization of Rho-dependent regions or *rut* sites while retaining the general distinction between RNA-end mapping and release evidence (Wang et al. 2023; Delaleau et al. 2025).

RhoTermPredict operationalizes *rut*-plus-pause logic by scanning a 78-nt C > G-rich region and downstream pause-like features (Di Salvo et al. 2019). Its reported F1 scores are about 0.7 in *E. coli* K-12 and 0.6 in *B. subtilis* 168 in positive and negative sequence-set tests; genome-wide candidates were assessed separately by RNA-seq read-count gradients. H-SELEX provides a complementary resource by mapping Rho-associated *rut* sites, while showing that in vivo engagement also depends on transcription, accessibility, growth context, and processing (Delaleau et al. 2025). Additional examples of Rho-associated signal features, perturbation evidence, and termination-zone measurements are summarized in Table S2.

Mechanistically, Rho-dependent termination is also route-flexible. Single-molecule and in vitro analyses support multiple routes, including catch-up and stand-by modes, with route usage shaped by the timing of Rho loading, translocation, and RNAP pausing (Song et al. 2022). Pause-extension experiments indicate that longer RNAP pauses preferentially benefit stand-by Rho-dependent termination over catch-up termination in the tested systems, linking termination efficiency to the lifetime of the kinetic window rather than to *rut* sequence alone (Song et al. 2023). NusA can also antagonize Rho in *E. coli* by competing for overlapping RNA-binding regions, delaying Rho access to nascent RNA (Qayyum et al. 2016). These examples make Rho-dependent termination a signal-processing problem: *rut* accessibility, factor competition, pause duration, and RNA processing all shape the final outcome.

Rho-dependent terminators are best described as *rut*-centered termination zones. They include an accessible, usually C-rich/G-poor RNA segment; a downstream region where RNAP pauses or slows; and a transcript context in which ribosomes or RNA structures do not block Rho before it acts. This distributed organization can silence pervasive transcription, enforce transcription-translation coupling, regulate leaders and riboswitches, and adapt to species-specific transcriptome architectures, even though measured outcomes may include a readthrough change, a reduced downstream RNA signal, a protected steady-state 3′ end, or a mapped *rut* peak.

## Archaeal U-rich terminators and cleavage-linked termination

Determinant weighting in archaeal termination is route-specific. Operationally, U-rich information can enter alternative rules: it can destabilize a susceptible elongation complex directly or be recognized within a factor- and cleavage-linked route. U-rich hybrid destabilization is most proximal to release in intrinsic-like routes, whereas U-rich recognition, aCPSF1/FttA action, Spt4/5 coupling, and cleavage contribute to cleavage-linked termination, for which the final release step remains less completely resolved. Factor perturbation, biochemical reconstitution, and structural studies support cleavage coupling more directly than RNA-end maps, while lineage, factor availability, sequence context, polymerase state, RNA processing, and condition modulate the observed outcome (Li et al. 2021; You et al. 2024).

### Intrinsic-like U/T-rich signals

Archaeal U-rich tracts can promote intrinsic-like release, recruit cleavage factors, or contribute to transcript-end maturation. Unlike canonical bacterial intrinsic terminators, they do not always require an upstream hairpin, although bacterial-like hairpin/U-tract terminators can remain active in archaeal systems (Maier and Marchfelder 2019; Wenck and Santangelo 2020). This diversity allows compact archaeal genomes to set transcript boundaries without simply recapitulating bacterial hairpin/U-tract mechanisms.

**Fig. 4:**
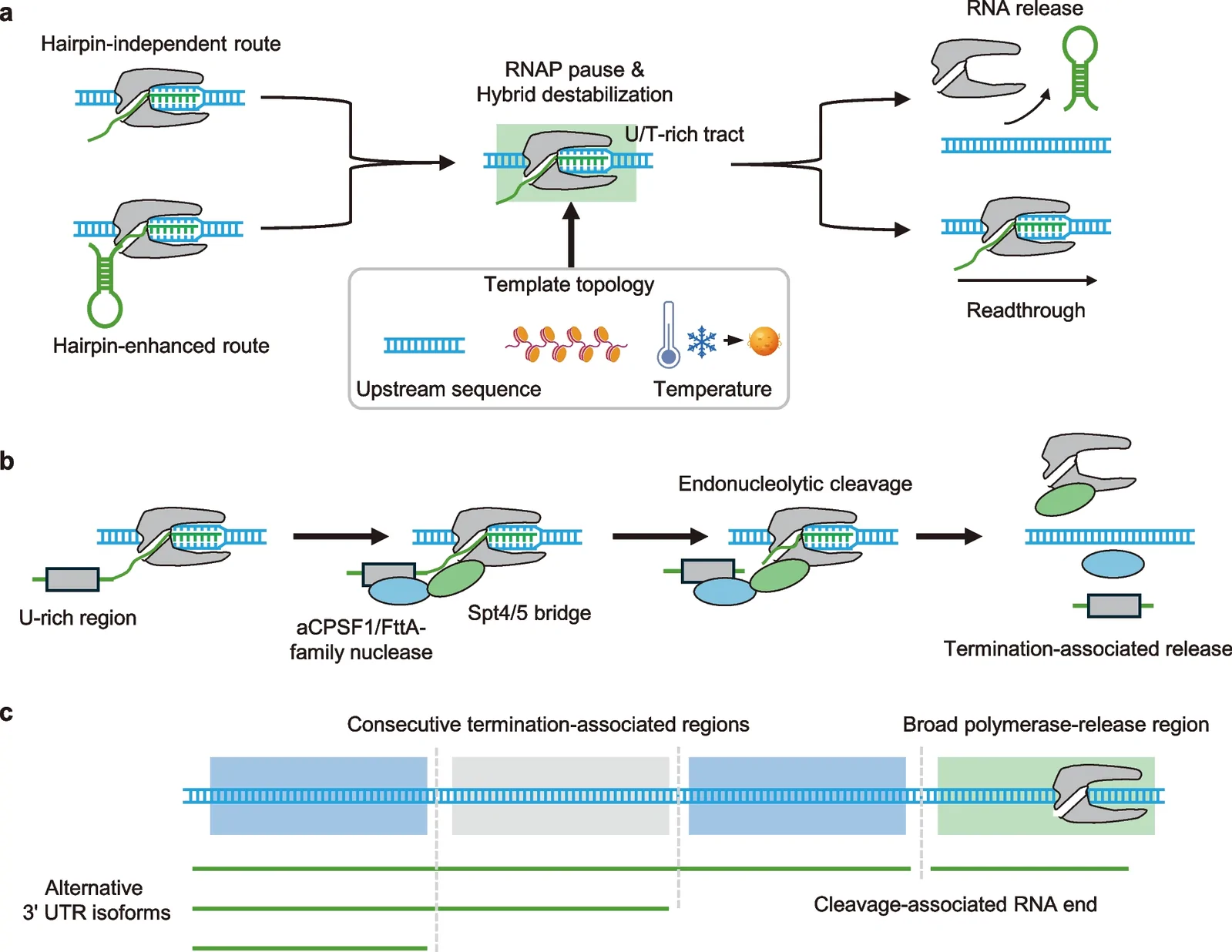
Archaeal U-rich and cleavage-linked termination grammars. **a** U/T-rich tracts can promote pausing, hybrid destabilization, and RNA release, with hairpin contributions varying among systems. **b** In cleavage-linked routes, aCPSF1/FttA-family nucleases act near U-rich RNA and can be coupled to the elongation complex through Spt4/5-family contacts. **c** Consecutive termination-associated regions can generate alternative 3′ UTR isoforms, and processed RNA ends can differ in position from the polymerase-release region

Foundational in vitro work established that archaeal RNAP can respond directly to T-rich sequences. *Methanothermobacter thermautotrophicus* RNAP terminated at bacterial-like intrinsic terminators and at synthetic or natural oligo-T-rich sequences without a recognizable upstream hairpin (Santangelo and Reeve 2006). In that system, a hairpin could enhance termination at bacterial-like terminators, but oligo-T-directed termination did not strictly require one. At the same time, the study showed that termination position and efficiency were sensitive to template topology, temperature, NTP conditions, T-tract length, and remote upstream sequences. These experiments support a U/T-tract-centered archaeal intrinsic signal, but their sensitivity to template and reaction conditions shows that oligo-T tracts are not autonomous stop signs. Figure 4a contrasts hairpin-independent with hairpin-enhanced archaeal termination configurations.

### Genome-wide 3′-end maps and transcript architecture

Genome-wide 3′-end mapping strengthened the case that U-rich tracts are a broad archaeal terminator-associated feature. Term-seq in *Methanosarcina mazei* and *Sulfolobus acidocaldarius* mapped RNA 3′ termini at single-base resolution and identified signatures dominated by uridine-rich blocks with little evidence for a universally required upstream RNA structure (Dar et al. 2016a). In *M. mazei*, the region immediately upstream of the mapped end was U-rich and G-depleted, whereas the *S. acidocaldarius* consensus contained proximal and distal U-rich blocks. These patterns support shared proximal U-rich logic with phylum-specific architecture. More than 30 percent of genes in the two species also produced alternative 3′ UTR isoforms through consecutive terminators, linking termination-associated end formation to archaeal transcript diversification (Fig. 4c).

*Haloferax volcanii* adds a third mapping perspective. Its 1,543 mapped RNA termini associated intergenic ends with a T-stretch motif and coding-region ends with a distinct AGATC-like signal (Berkemer et al. 2020). Reporter tests supported termination activity for selected candidates, and multiple 3′ UTRs again suggested regulatory potential. The archaeal-specific caution is that cleavage-linked pathways can place processing and termination-associated ends close together; mechanistic assignment is strengthened when RNA-end evidence is paired with factor perturbation, reporter assays, or nascent-transcription evidence.

### aCPSF1/FttA-linked cleavage and other factor-dependent routes

The major recent change in the archaeal field is the emergence of cleavage-linked termination mediated by CPSF73-family ribonucleases. In *Methanococcus maripaludis*, depletion of aCPSF1 caused genome-wide transcription termination defects and transcriptome disorder, while Term-seq indicated that transcripts primarily terminate downstream of a uridine-rich motif where aCPSF1 performs endoribonucleolytic cleavage (Yue et al. 2020). The endoribonuclease activity was required for in vivo termination, and distant aCPSF1 orthologs from Lokiarchaeota and Thaumarchaeota could functionally replace *M. maripaludis* aCPSF1 in the tested system. Together, the phylogenetic distribution and ortholog-replacement experiments support a conserved role for aCPSF1 in the tested archaeal lineages.

The relationship between U-rich tracts and aCPSF1 was clarified by work showing that aCPSF1 cooperates with terminator U-tracts to determine termination efficacy (Li et al. 2021). In *M. maripaludis*, efficacy correlated positively with the number of U4 tracts upstream of primary termination sites, and aCPSF1 depletion reduced it genome-wide. In vitro binding assays and in vivo reporters showed that the N-terminal KH domains recognize terminator U-tracts, while nuclease activity is required for high efficacy. U-rich RNA recognition, factor binding, and cleavage can therefore strengthen weak intrinsic-like signals.

In reconstituted in vitro assays, FttA, a CPSF73-family homolog, can terminate archaeal transcription by cleaving nascent RNA from stalled or vulnerable elongation complexes (Sanders et al. 2020). Later cryo-EM work on a *Thermococcus kodakarensis* FttA-Spt4/5-TEC pre-termination complex showed RNA leaving the TEC exit channel and being positioned toward the FttA catalytic center; cleavage was mapped at a defined distance from the RNA 3′ end, and Spt5 bridged FttA to the TEC (You et al. 2024). This structure explains how a ribonuclease can be coupled physically to the archaeal elongation complex and how Spt5 can stimulate FttA-dependent termination. U-rich RNA recognition, nuclease engagement, Spt4/5-linked coupling, cleavage, and termination-associated release form connected but distinct stages (Fig. 4b). Compared with bacterial Rho, aCPSF1- and FttA-family proteins are also associated with factor-assisted, RNA-engaging termination routes and NusG/Spt5-family contacts, but they are cleavage-linked ribonucleases rather than ATP-dependent helicase/translocases. Additional examples of archaeal U-rich, cleavage-linked, and factor-associated termination are summarized in Table S3.

Other archaeal factors and transcriptomic data broaden this landscape without defining a canonical downstream terminator. Eta disrupts stalled or arrested elongation complexes in vitro, and virus or host regulators can alter RNAP through channel occlusion, factor mimicry, or trigger-loop/bridge-helix interference (Walker et al. 2017; Pilotto and Werner 2022). Direct RNA sequencing captures isoform and UTR complexity but, because of truncation, processing, degradation, and sparse sampling, identifies candidate termination or processing regions rather than release mechanisms (Mattick et al. 2024).

Protein-family relationships linking aCPSF1/FttA to CPSF73 and Spt5 to NusG support shared molecular ancestry at the component level, but they do not demonstrate complete-pathway homology (Sanders et al. 2020; You et al. 2024). Archaeal systems are therefore best treated as distinct solutions that illuminate evolutionary connections without serving as a simple intermediate between bacterial and eukaryotic termination.

## Eukaryotic nuclear transcription termination

PAS recognition and cleavage and polyadenylation (CPA) factor-mediated cleavage initiate RNAPII termination but are not themselves the polymerase-release step. Operationally, PAS and auxiliary elements are spatially organized for CPA recognition and cleavage, after which Xrn2/Rat1 action, allosteric change, elongation kinetics, and chromatin condition the probability and position of downstream RNAPII release. Post-cleavage pausing and allosteric change, together with Xrn2/Rat1-dependent degradation and catch-up, act more proximally to release within a downstream window; PAS strength, cleavage-site context, elongation state, chromatin, and cellular condition modulate their effectiveness. Biochemical, factor-perturbation, and nascent-transcription evidence jointly support this ordering, whereas poly(A)-site and steady-state readthrough measurements resolve related but distinct outcomes (Eaton and West 2018, 2020). Beyond this dominant PAS-centered pathway, Pol I, Pol III, and transcript-class-specific RNAPII units use distinct sequence-, factor-, or processing-coupled termination grammars.

### PAS grammar and cleavage/polyadenylation

PAS-centered RNAPII termination is initiated at a CPA region rather than at a bacterial-style stop element. The core cis element is usually AAUAAA or a related hexamer, positioned upstream of the cleavage site and read together with auxiliary upstream and downstream sequence elements (Neve et al. 2017; Kumar et al. 2019; Rodríguez-Molina et al. 2023). The nascent RNA is cleaved and polyadenylated to form an mRNA 3′ end, while RNAPII terminates later within a downstream window. RNA maturation and polymerase release are therefore coupled but distinct events. Reconstituted and structural studies identify WDR33 and CPSF30 as direct AAUAAA readers, with CPSF160 organizing the recognition surface (Schönemann et al. 2014; Clerici et al. 2017, 2018; Sun et al. 2018).

**Fig. 5:**
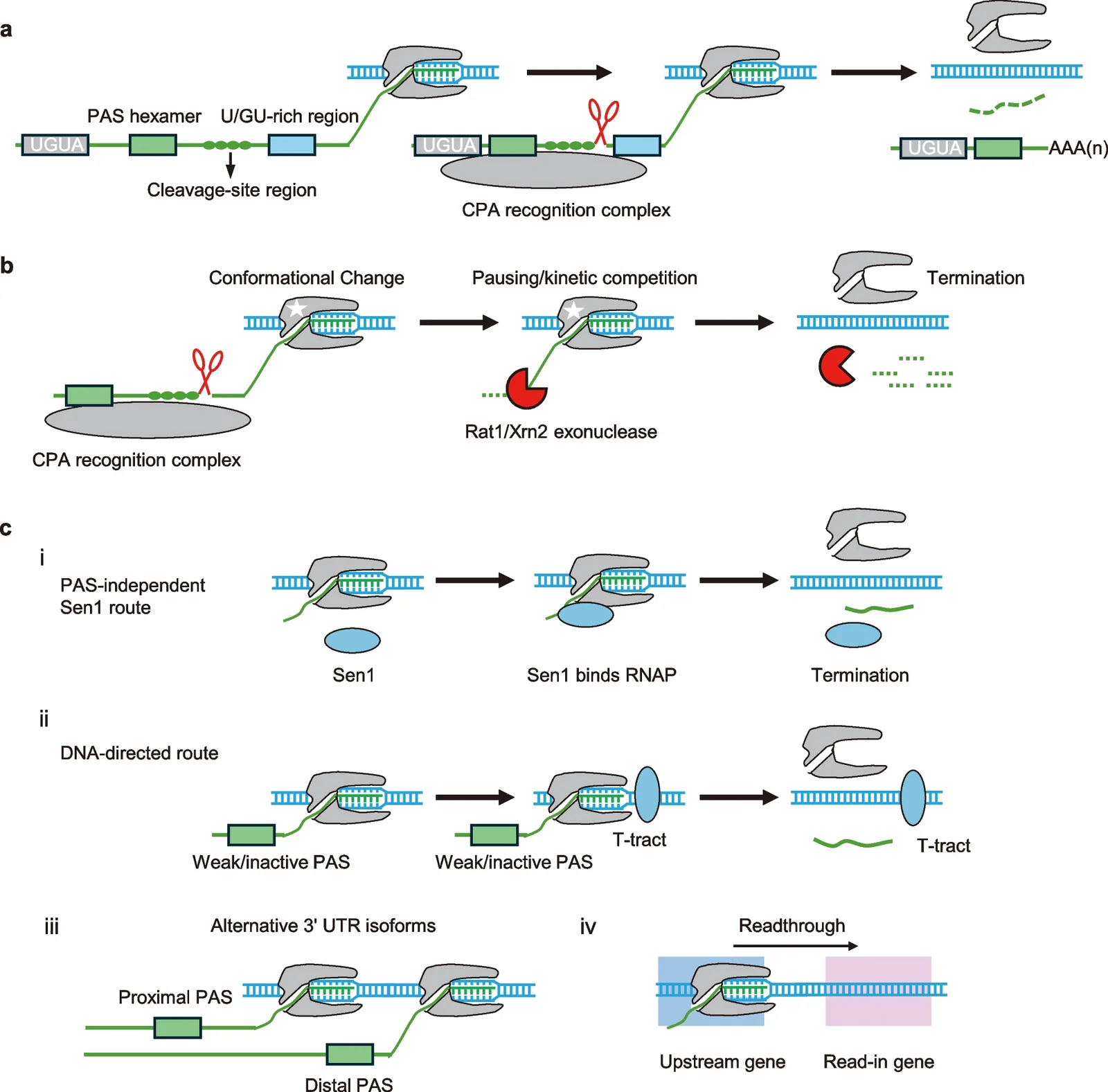
PAS-centered RNAPII termination and downstream termination windows. **a** PAS and auxiliary elements recruit cleavage and polyadenylation machinery to generate the mature upstream RNA and downstream fragment. **b** Rat1/Xrn2 activity, allosteric change, elongation control, and chromatin act across a downstream termination window. **c** (i) Sen1 supports a PAS-independent termination route. (ii) T-rich DNA elements can support DNA-directed termination in specific contexts. (iii) Alternative PAS choice generates distinct 3′ UTR isoforms. (iv) Upstream termination failure can produce readthrough and read-in transcription

PAS recognition depends on more than the hexamer. In metazoan systems, downstream U-rich or GU-rich elements are recognized by CstF-related machinery, while upstream auxiliary elements and UGUA-like motifs can recruit additional factors such as CFIm; together these elements help define cleavage position and site strength (Neve et al. 2017; Sun et al. 2020). Quantitative sequence-function work supports this combinatorial view. A massively parallel reporter study showed that PAS variants and nearby sequence context can tune expression over a wide range, although it did not directly measure chromatin-associated RNAPII termination (Vainberg Slutskin et al. 2019). PAS strength therefore reflects hexamer identity, flanking motifs, spacing, and cellular factor availability rather than AAUAAA alone. These features influence cleavage/polyadenylation while the mature RNA product, downstream cleavage fragment, and continuing RNAPII remain distinct (Fig. 5a).

### From PAS cleavage to RNAPII release

The classical torpedo model proposes that PAS cleavage creates a downstream RNA with a 5′ phosphate that can be degraded by Rat1/Xrn2, allowing the exonuclease to catch RNAPII and promote release. Genome-wide yeast work and human mechanistic studies support an important role for Rat1/Xrn2-family activity in termination downstream of polyadenylation sites (Baejen et al. 2017; Eaton and West 2018). RNAPII termination also involves allosteric and kinetic changes after PAS recognition, including CPA-dependent changes in the elongation complex, Spt5/CTD-linked regulation, polymerase slowing, pausing, and chromatin or R-loop context (Eaton and West 2020; Rodríguez-Molina et al. 2023). The evidence best supports a coupled allosteric/torpedo mechanism in which PAS recognition and cleavage help make RNAPII termination-prone, and Xrn2 acts efficiently when polymerase has slowed enough for termination to occur. PAS cleavage, Xrn2/Rat1 activity, elongation kinetics, and chromatin context together shape termination efficiency within a broad downstream termination window (Fig. 5b).

Genome-wide measurements have made this window concept practical. In yeast, termination windows can be inferred between the last poly(A) site and the downstream drop in nascent or newly synthesized RNA signal, and factor perturbations can shift or lengthen that window (Baejen et al. 2017). In mammalian cells, readthrough analyses similarly show that loss or weakening of processing and termination factors produces extended downstream transcription rather than a single alternate endpoint (Roth et al. 2020; Caldas et al. 2024). Together, these datasets place cleavage-site choice, downstream loss of nascent transcription, and accumulated readthrough RNA at different positions within the RNAPII termination window.

Chromatin can further shape PAS choice and the downstream termination window. Depletion of yeast histone chaperones FACT and Spt6 shifted poly(A)-site usage upstream, whereas histone depletion shifted usage downstream, consistent with effects of chromatin on RNAPII elongation rate and processivity (Geisberg et al. 2024). Because poly(A)-site shifts are indirect proxies for elongation kinetics, this evidence supports chromatin-dependent bias in cleavage and termination behavior rather than a model in which chromatin alone encodes a terminator.

### Alternative PAS choice, readthrough, and non-canonical RNAPII termination

Alternative PAS choice, readthrough, and non-canonical release routes operate alongside the canonical protein-coding-gene PAS/CPA pathway. In a polyadenylation-independent context, recent cryo-EM and in vitro work on Sen1-bound RNAPII complexes supports a model in which Sen1 binds RNAPII, contacts upstream RNA, and uses ATP-dependent 5′-to-3′ translocase activity to pull RNA and destabilize the elongation complex (Rengachari et al. 2025) (Fig. 5c, i).

DNA-directed termination provides a second non-canonical example. Studies in yeast and mammalian systems report that T-rich DNA elements can contribute to RNAPII termination in cleavage-independent or cleavage-complementary contexts (Han et al. 2023; Davidson et al. 2024). The mammalian evidence is strongest for promoter-proximal regions and short transcription units such as snRNA-related contexts, whereas Xrn2-dependent termination remains dominant downstream of most protein-coding genes (Davidson et al. 2024). These findings identify contexts in which RNAPII responds to terminator-like DNA features without replacing the canonical PAS/CPA/Xrn2 pathway (Fig. 5c, ii).

Alternative polyadenylation (APA) through PAS choice changes 3′ UTR length and sometimes coding output; its use varies with processing factors, RNA-binding proteins, and cell state, altering post-transcriptional regulation (Tian and Manley 2017; Mayr 2019). Cross-species resources and plant functional studies connect APA and PAS choice to FY, terminator features, and processing-factor perturbations (Wang et al. 2018; Yu et al. 2019; Herrmann et al. 2020; Zhu et al. 2020; Yang et al. 2021; Mo et al. 2021; Gorjifard et al. 2024; Jin et al. 2025). These data distinguish coordinated PAS recognition and cleavage from downstream RNAPII release (Fig. 5c, iii).

Readthrough and downstream-of-gene (DoG) transcription are downstream outcomes when termination or processing is altered: stress can induce many human genes to produce long readthrough transcripts, and atlas data indicate that readthrough also occurs in healthy tissues in association with genomic and termination-region features (Vilborg et al. 2015; Caldas et al. 2024) (Fig. 5c, iv). ARTDeco enables RNA-seq-based quantification of such events, while proposed DoG functions such as miRNA sponging or chromatin regulation require locus-specific perturbation tests (Roth et al. 2020; Rosa-Mercado and Steitz 2022). In yeast, defective cleavage or termination can extend transcripts into downstream genes, reduce splicing efficiency, and expose cryptic splice junctions, showing that termination windows protect neighboring transcript architecture as well as defining downstream polymerase-release regions (Barr et al. 2025). Together, these routes show that RNAPII termination is PAS-centered in initiation but window-shaped in outcome: PAS and auxiliary elements direct cleavage and polyadenylation, while Xrn2-compatible RNA, elongation kinetics, pausing, chromatin, and processing context shape downstream release (Table S4).

### Other nuclear RNA polymerases and transcript-class-specific RNAPII termination

Eukaryotic nuclear termination extends beyond PAS-centered RNAPII transcripts. Pol I and Pol III use compact sequence- or factor-bound signals, whereas specialized RNAPII transcription units use transcript-class-specific routes. These systems share constraints imposed by multisubunit RNAP and kinetic competition with elongation, but use distinct cis elements, readers, spacing, processing connections, and release windows (Table S4).

Pol I termination combines DNA-bound terminator factors with pausing and, in at least one system, a conserved RNA hairpin. In defined-scaffold assays, a roadblock plus T-rich region releases Pol I RNA inefficiently without an upstream hairpin; nearly all hairpin-containing scaffold RNA was released by the earliest measurable 20-s time point (Nielsen and Zenkin 2025). Genome-wide and biochemical analyses in budding yeast indicate multiple routes: Rnt1 cleavage, Rat1, and Rpa12 contribute to canonical termination, whereas TRAMP/Rat1-associated events participate in start-proximal surveillance, with premature termination modeled at about 10% rather than directly counted (Petfalski et al. 2025). Pol I therefore integrates factor-bound DNA signals, pausing, RNA structure, and processing or surveillance context.

Pol III termination is compact and largely polymerase intrinsic. Cryo-EM structures of budding-yeast pre-termination complexes show that the C128 lobe recognizes the non-template poly(dT) tract and stabilizes pausing before release (Girbig et al. 2022). Biochemical and transcriptomic work shows that T tracts are necessary but not always sufficient, nearby RNA hairpins can enhance weak terminators, and Sen1 can act downstream as a fail-safe (Xie et al. 2022). The final clamp opening, hybrid collapse, and release sequence remains partly modeled, and these budding-yeast findings may not apply to every Pol III system.

Specialized RNAPII routes extend the Sen1 and T-rich examples in Sect. 6.3. Single-molecule reconstitution showed that Sen1 forms a one-molecule intermediate on stalled Pol II, releases RNA in an ATP-coupled step, and leaves heterogeneous DNA-associated Pol II states; RNA translocation was estimated at about 80 nt/s (Xiong et al. 2024). Integrator associates with the RNAPII CTD, is recruited to U1/U2 snRNA genes for precursor 3′-end processing, and can be targeted to promoters through transcription-factor contacts (Baillat et al. 2005; Sabath et al. 2024). Replication-dependent histone transcripts use overlapping PAS and histone downstream element (HDE) information to recruit canonical or histone-specific processing complexes; PAS disruption selectively blocks canonical recruitment (Ielasi et al. 2022). Together, these examples span recruitment, processing, and RNA-release endpoints rather than direct Pol II-DNA dissociation.

### Pathophysiological consequences of altered 3′-end processing and RNAPII termination

Human disease can involve cis-acting PAS variants, dysregulated CPA or termination-related factors, and broader APA remodeling. These categories affect overlapping stages of RNA 3′-end formation but provide different evidence for defective polymerase release. Disease-associated APA shifts may reflect altered PAS recognition, cleavage, isoform stability, or cell-state composition; attribution to RNAPII termination requires downstream-transcription or release measurements (Tian et al. 2026). Table 3 therefore reports the measured process and interpretation limit for each example.

**Table 3:** Representative disease and pathophysiological contexts associated with altered 3′-end processing, alternative polyadenylation, or RNAPII termination-related factors

Alteration class	Gene/factor and process	Disease context	Evidence and interpretation
Cis-acting somatic variants	Tumour-suppressor PASs (XPA); PAS recognition and readthrough	Pan-cancer and colorectal-cancer analyses	PCAWG + XPA minigene (Kainov et al. 2024); recurrent PAS disruption; no release
Cis-acting PAS variants	Polyadenylation hexamers; APA and transcript extension	Human variant classification and Mendelian-disease assessment	PAS annotation + ClinSeq (Shiferaw et al. 2023); hexamer score not pathogenic
Trans-regulatory factor abundance	CPSF1; poly(A)-site selection, abundance, glycolytic output	Prostate cancer	Patient data + CPSF1 knockdown (Tietz et al. 2025); no release measured
Trans-regulatory factor interaction	XRN2-Sam68; alternative PAS choice and cell-cycle progression	Prostate cancer	Interaction, CLIP, rescue (Pieraccioli et al. 2022); APA role; ≠ torpedo release
Trans-regulatory CPA-complex remodeling	MAGE-A11-HUWE1-PCF11-CFIm25; CPA composition and 3′-UTR shortening	Lung squamous, ovarian, and other MAGE-A11-positive cancers	Cohort, biochemical, xenograft (Yang et al. 2020); UTR shortening ≠ release
Disease-causing helicase variants	SETX; Sen1-family RNA-helicase function	Ataxia with oculomotor apraxia type 2	Linkage and segregation (Moreira et al. 2004); causal SETX variants; termination defect not tested
Cell-state-associated APA feedback	CFIm25-PCBP1; 3′-UTR shortening, translational control, differentiation	Human macrophage differentiation	3′-end/RNA-seq, RIP (del Pilar Mendoza-Martín et al. 2026); APA circuit; no direct release defect
Disease-associated APA program	Immune-related APA; recurrent 3′-UTR lengthening	Systemic lupus erythematosus	RNA-seq/DaPars2 (Chen et al. 2026); patient-stratifying APA; CPA or release causality not tested

The table distinguishes cis-acting variants, trans-regulatory abnormalities, and cell-state-associated APA programs by affected process, evidence basis, and interpretation. APA, cleavage-site readthrough, transcript extension, and direct polymerase release are treated as distinct experimental endpoints

Cis-acting variants directly connect a local signal to an altered RNA outcome. Pan-cancer analysis identified recurrent somatic variants predicted to weaken cleavage and polyadenylation signals in tumour-suppressor genes. For an XPA AATAAA-to-GATAAA variant, a minigene assay showed increased cleavage-site readthrough and reduced reporter expression, supporting a functional PAS defect without locating RNAPII release (Kainov et al. 2024). A genome-wide catalogue likewise associated a subset of variants in predominant polyadenylation hexamers with APA or transcript extension. Such catalogues can prioritize variants for clinical interpretation, although sequence position and computational score alone do not establish pathogenicity (Shiferaw et al. 2023).

Trans-regulatory abnormalities can alter many 3′ ends simultaneously. In prostate-cancer models, CPSF1 depletion increased downstream intergenic poly(A)-site use, reduced expression of many transcripts, and impaired glycolytic output (Tietz et al. 2025). XRN2 and Sam68 regulate an overlapping APA program that promotes proximal PAS use and cell-cycle progression; rescue by exonuclease-deficient XRN2 separates this APA function from exonuclease-dependent termination (Pieraccioli et al. 2022). MAGE-A11 also redirects HUWE1 toward PCF11, reduces CFIm25 association with the 3′-end-processing complex, and promotes proximal PAS use and tumour growth (Yang et al. 2020). These findings concern CPA activity and isoform choice rather than polymerase-release position.

Termination-related factor families also connect this field to neurological and immune-cell phenotypes. Biallelic SETX mutations cause ataxia with oculomotor apraxia type 2, linking disease to a Sen1-family helicase, although the defining genetic study did not establish a specific causal termination defect (Moreira et al. 2004). During macrophage differentiation, APA shortens the CFIm25 3′ UTR, reduces PCBP1-mediated repression, and increases CFIm25 protein, forming a regulatory feedback circuit rather than an established immune-disease or direct-release mechanism (del Pilar Mendoza-Martín et al. 2026). Multi-cohort RNA-seq analysis of systemic lupus erythematosus identified recurrent 3′-UTR lengthening and immune-related APA patterns that stratified patients, but did not identify a causal CPA-factor defect or altered RNAPII release (Chen et al. 2026). These examples distinguish disease association and RNA-end regulation from direct RNAPII-release evidence.

## Cross-domain convergence and divergence of termination signal grammars

Across bacteria, archaea, and eukaryotes, termination converts a processive elongation complex into a release-prone or processing-coupled state. The conserved multisubunit RNAP core imposes shared physical constraints, but it does not prescribe one signal architecture or termination machinery (Minakhin et al. 2001). Cross-domain comparison therefore asks how common kinetic and biochemical problems are solved within different cellular organizations.

### Shared kinetic and biochemical constraints

Elongation and termination compete for the same elongation complex. Productive termination therefore requires signal recognition or factor action before RNAP escapes the relevant kinetic window, together with an elongation state susceptible to pausing, destabilization, cleavage coupling, or release. Signal recognition, RNA processing, and polymerase release need not occur at the same position or time. The recurring constraint is coordination among cis-acting signal information, molecular machinery, polymerase state, and cellular context, not a universal clock or release coordinate.

**Fig. 6:**
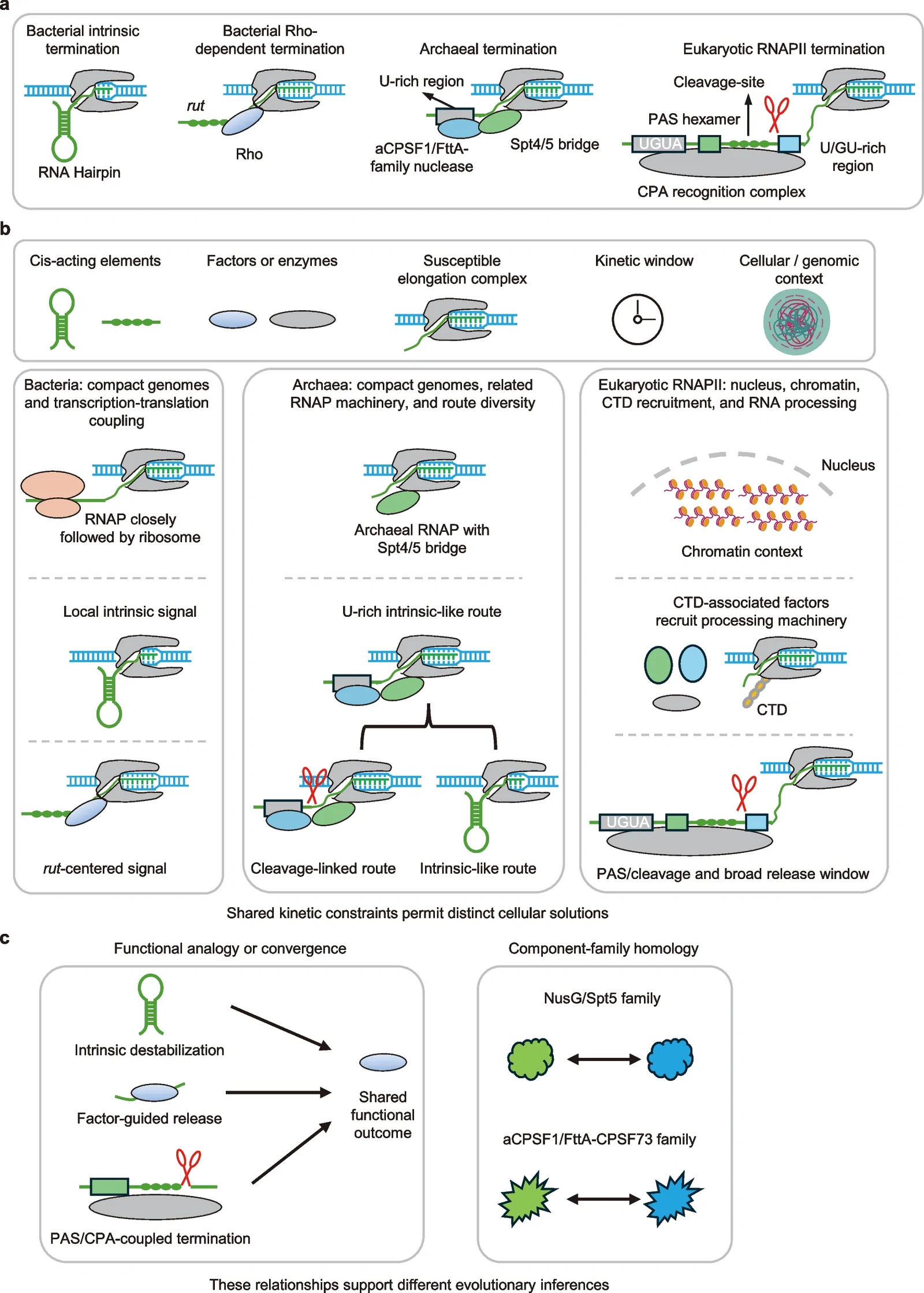
Cross-domain convergence, divergence, and evolutionary interpretation. **a** Intrinsic terminators use a compact hairpin/U-rich module; Rho-dependent termination spans an upstream *rut*-centered RNA region and a downstream termination zone; archaeal termination includes U-rich intrinsic-like and cleavage-linked alternatives; and RNAPII termination couples an upstream PAS/CPA region to a broad downstream release window. **b** All systems require cis-acting signal features, a susceptible elongation complex, molecular machinery acting before escape, and a permissive context, but bacterial transcription-translation coupling, archaeal route diversity, and eukaryotic nuclear, chromatin, CTD, and RNA-processing organization favor different spatial and factor dependencies. **c** Functional analogy or convergence and component-family homology support different evolutionary inferences and do not define a linear transition among extant pathways

Bacterial intrinsic terminators concentrate much of this logic in a local hairpin/U-tract module: hairpin formation, a weak rU-dA hybrid, and terminator-proximal pausing can be encoded within a short region. Compactness nevertheless does not imply context independence, because folding pathway, local sequence, RNAP state, and accessory factors tune efficiency (Ray-Soni et al. 2016; Roberts 2019; You et al. 2023). Rho-dependent termination distributes the corresponding requirements across an accessible *rut* region, translation status, RNA structure, Rho translocation, and a downstream pause or slowing zone. Archaeal systems combine U-rich intrinsic-like destabilization with lineage-dependent cleavage-linked routes, whereas RNAPII systems couple PAS recognition and cleavage/polyadenylation to a downstream release window shaped by Xrn2/Rat1, elongation kinetics, chromatin, and RNA-processing context (Neve et al. 2017; Wenck and Santangelo 2020; Song et al. 2022; Rodríguez-Molina et al. 2023) (Fig. 6a). Pol I, Pol III, and specialized RNAPII pathways add further examples: factor-bound and hairpin-assisted Pol I signals, polymerase-intrinsic poly(dT) recognition by Pol III, and processing-coupled or factor-dependent RNAPII routes such as Integrator, NNS/Sen1, and replication-dependent histone transcripts (Table 1; Table S4). Collectively, these architectures are distinct solutions to the shared problem of making an elongation complex termination-prone.

The common kinetic constraint is realized differently in each system. Intrinsic termination relies mainly on RNAP and nascent-RNA energetics; Rho must engage exposed RNA and use ATP-dependent translocation before the complex escapes; archaeal U-rich recognition can act through intrinsic-like or cleavage-linked routes; and RNAPII processing and exonuclease action operate across a broad downstream window (Schwalb et al. 2016; Eaton and West 2020; Li et al. 2021; You et al. 2023, 2024; Molodtsov et al. 2023). Figure 6b compares these shared constraints without collapsing their molecular execution.

### Polymerase evolution, cellular organization, and lineage-specific machinery

RNA polymerase evolution constrains how termination grammars can vary. All multisubunit RNAPs share a conserved core architecture, and bacterial subunit omega and eukaryotic RPB6 are sequence, structural, and functional homologs that promote assembly (Minakhin et al. 2001). Archaeal and eukaryotic RNAPs share similar subunit architectures, providing a basis for the related elongation-factor families observed in these lineages (Best and Olsen 2001). RNAPII CTD elaboration and the specialization of Pol I and Pol III then allow transcript-class-specific coupling to processing and termination. NusG/Spt5-family factors illustrate how a conserved elongation-factor family can be recruited into distinct termination contexts, from bacterial Rho coupling to archaeal FttA bridging and eukaryotic Spt5-linked exoribonucleolysis (You et al. 2024; Kuś et al. 2025). These component relationships support evolutionary comparison without establishing complete-pathway homology.

Mechanistic divergence can be related to cellular organization without treating present-day differences as a reconstructed historical sequence. In bacteria, compact genome organization, short intergenic regions, and direct transcription-translation coupling are consistent with rapidly interpreted local intrinsic modules and *rut*-centered zones whose accessibility changes with ribosome occupancy. Rho variation can alter this coupling under stress, but its proposed evolutionary role remains lineage- and condition-dependent (Johnson et al. 2020; Worthan et al. 2025). Archaea combine compact genomes with RNAP and elongation-factor families related to eukaryotic counterparts, allowing U-rich intrinsic-like and cleavage-linked solutions to coexist rather than placing archaea on a simple path from bacterial to eukaryotic termination (Wenck and Santangelo 2020; You et al. 2024).

In eukaryotes, nuclear separation of transcription from translation, chromatin organization, CTD-dependent recruitment, and extensive RNA processing may have favored spatial separation among PAS recognition, cleavage, and polymerase release. This interpretation is consistent with kilobase-scale RNAPII termination windows, chromatin-sensitive PAS choice, and direct coupling of Spt5-family elongation factors to Xrn2-dependent release, but it does not establish that broad windows evolved through a direct transition from bacterial intrinsic hairpins (Schwalb et al. 2016; Geisberg et al. 2024; Kuś et al. 2025).

### Homology, convergence, and limits of evolutionary inference

Functional similarity and evolutionary relatedness require different evidence. Similar kinetic logic can support functional analogy or convergence, whereas homology between aCPSF1/FttA and CPSF73-family nucleases or between NusG and Spt5 supports shared ancestry at the component-family level. Homologous components can be recruited into different pathways, and unrelated factors can solve comparable release problems. Cross-domain synthesis therefore supports conserved polymerase constraints, selected component homologies, and divergent cellular solutions, but not a single linear trajectory for complete termination pathways (Fig. 6c) (Porrua et al. 2016; Sanders et al. 2020; You et al. 2024; Kuś et al. 2025). Table 1 summarizes the mechanistic comparison, while Table 2 separates the measurements from the conclusions they support.

## Computational biology and machine learning for terminator discovery, prediction, and design

**Fig. 7:**
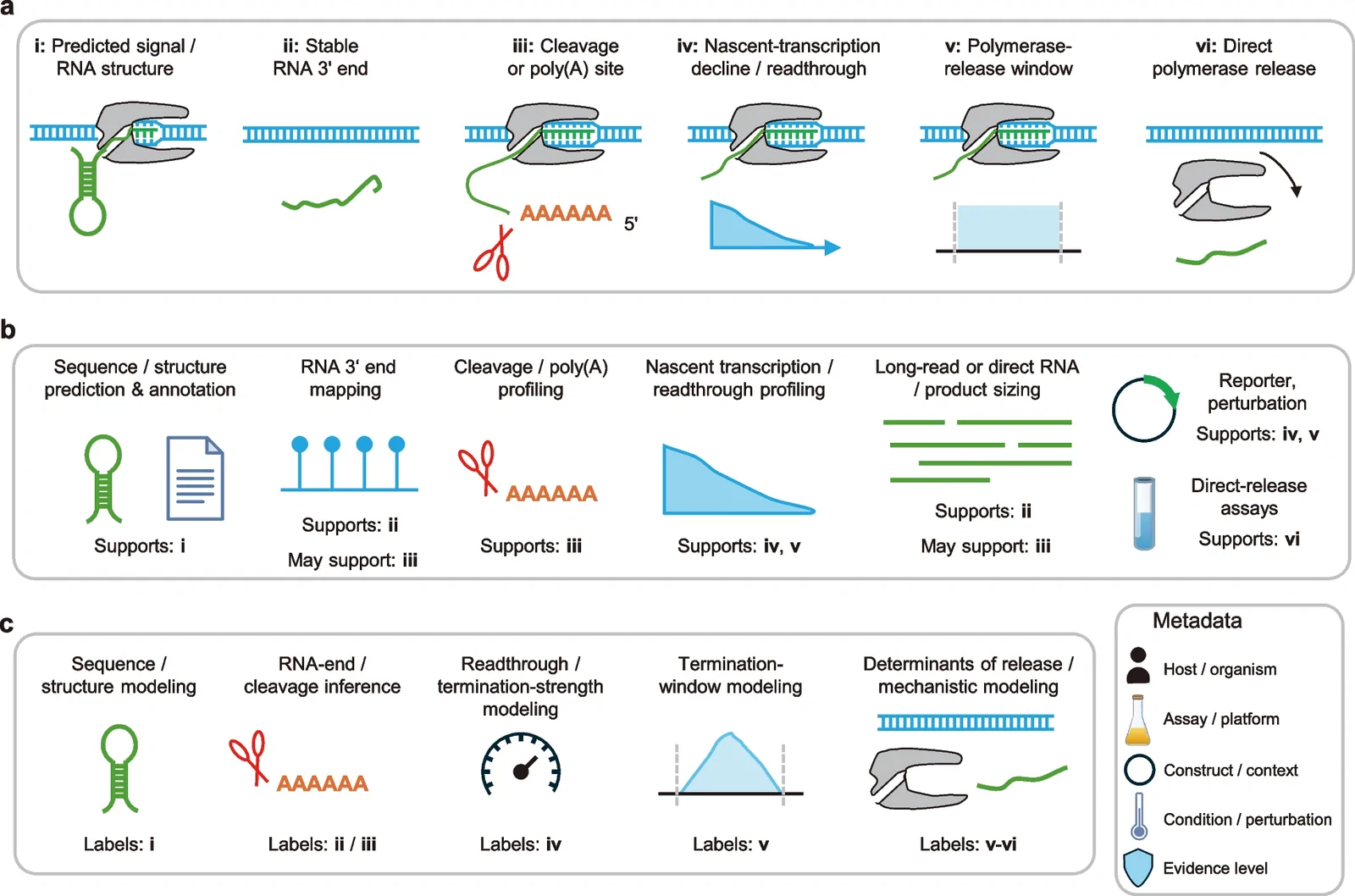
Experimental and computational methods resolve distinct, rather than interchangeable, termination-related observables and endpoints. **a** Predicted signal features, stable RNA 3′ ends, cleavage or poly(A) sites, nascent-transcription decline or readthrough, polymerase-release windows, and direct release represent distinct prediction outputs, experimental observables, and mechanistic endpoints. **b** Prediction and annotation, RNA-end or cleavage-site mapping, nascent-transcription profiling, long-read or direct RNA sequencing, reporter and perturbation assays, biochemical reconstitution, single-molecule assays, and direct-release measurements support different inferences. **c** Computational tasks require endpoint-matched labels, metadata, and experimental validation; computational claims inherit the evidential scope and limitations of their experimental labels. Long-read and direct RNA approaches provide RNA-product length information, which does not identify a polymerase-release position or window

Experimental and computational methods become comparable when they are organized by the predicted features, experimental observables, or biological endpoints they address rather than by platform name. Sequence or structure prediction identifies candidate signals; Term-seq, Rend-seq, 3′-end sequencing, and poly(A)-site atlases map stable RNA ends or cleavage sites; nascent-transcription and readthrough profiling localize changes in polymerase progression; and reporters, perturbations, biochemical reconstitution, and direct-release assays test function or mechanism at different levels (Fig. 7a, b; Table 2). Long-read and direct RNA approaches additionally provide RNA-product length information, while single-RNA sizing can resolve alternative product lengths in a defined transcription system. Product length is not itself equivalent to a polymerase-release position or release window, and these RNA-product measurements do not establish polymerase release unless dissociation is independently observed (Tarnowski and Gorochowski 2022; Patiño-Guillén et al. 2024).

**Fig. 8:**
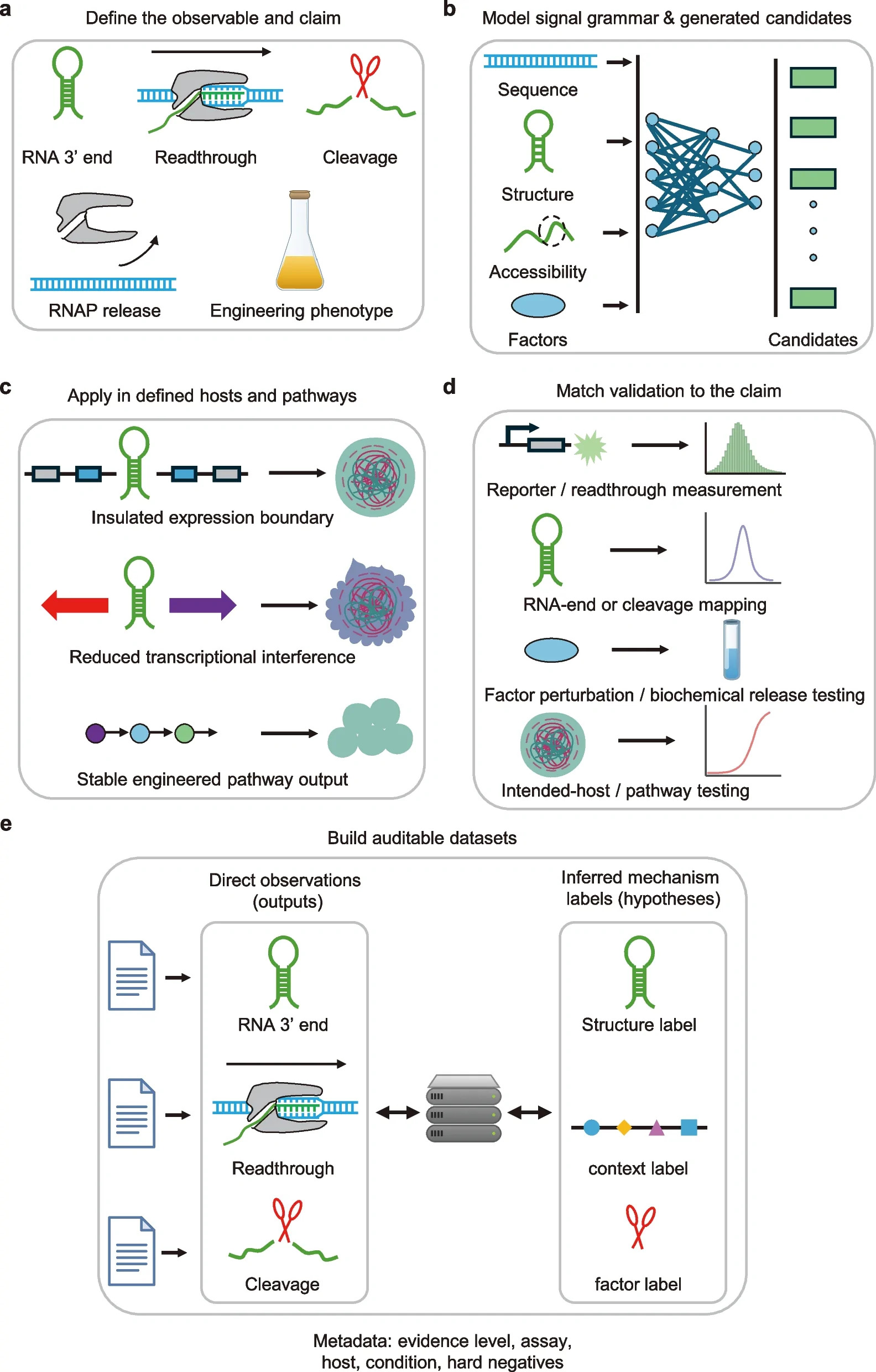
Claim-matched workflow for terminator discovery, validation, and design. **a** The workflow begins by defining the measurement to be predicted or engineered, distinguishing RNA-end formation, cleavage, readthrough, polymerase release, and engineered-construct performance. **b** Models use sequence, RNA structure and accessibility, spacing, factors, and host or cellular context to rank or generate candidates. **c** Intended applications are defined in the relevant host or pathway, including expression-boundary insulation, reduced transcriptional interference, and stable engineered pathway output. **d** Validation is matched to the claim through reporter or readthrough measurements, RNA-end or cleavage mapping, factor perturbation or biochemical release testing, and intended-host or pathway testing. **e** Direct observations, inferred labels, assay and context metadata, and hard negatives are retained in auditable datasets for model refinement. Performance is retested when host, construct, or condition changes

Computational tasks inherit these distinctions among predicted features, experimental observables, and mechanistic endpoints. Motif prediction, RNA-end or cleavage-site prediction, readthrough or termination-window prediction, reporter-efficiency prediction, and release-mechanism inference require matched training labels and validation (Fig. 7c). Candidate discovery, benchmarking, and sequence design additionally depend on host, assay, construct, condition, and evidence metadata. Figure 8 then organizes these requirements as a claim-matched workflow from defining the measurement and generating candidates to intended application, experimental validation, and auditable dataset refinement.

### Training labels, measurements, and candidate annotation

Computational discovery of termination signals depends first on the training or reference label. A bacterial intrinsic terminator label may mean a predicted hairpin/U-tract, a stable Term-seq 3′ end with TransTermHP support, a reporter-measured efficiency value, or a curated database entry. A Rho-dependent label may mean Rho-inhibitor readthrough, a *rut*-like sequence, an RNA-seq gradient, or a mapped Rho utilization site. A eukaryotic label may mean a poly(A) site, a cleavage site, tissue-specific PAS dominance, readthrough signal, or RNAPII termination window. These labels are biologically related but represent different measurements. The central challenge for machine-learning models is to match the prediction task to training or reference data that measure the same outcome (Wang et al. 2018; Herrmann et al. 2020; Roth et al. 2020; Kosiński et al. 2025) (Figs. 7c and 8a).

Bacterial candidate discovery spans intrinsic and Rho-dependent tasks that differ in what their labels represent. For intrinsic terminators, sequence-composition classifiers and CNN ensembles such as BacTermFinder can identify terminator-like regions across bacteria with different GC contents, but their outputs are candidate annotations, not mechanistic endpoint calls; performance declines with GC content, and fixed windows can miss upstream Rho-utilization information (Feng et al. 2019; Taheri Ghahfarokhi and Peña-Castillo 2025). For Rho-dependent termination, mechanism-specific scanners such as RhoTermPredict encode *rut*-plus-pause models and achieve F1 values around 0.6–0.7 in *E. coli* and *B. subtilis*, but validation based on RNA-seq read-count gradients can be influenced by gene-expression level and transcription-translation coupling, so these tools are best used to generate hypotheses, not final annotations (Di Salvo et al. 2019). Resource databases add complementary data layers: INTERPIN provides predicted hairpins across 12,745 bacterial genomes, and TERMITe identifies reproducible Term-seq ends with optional site-specific efficiency estimates; their predictions and RNA-end calls should retain their evidence type when used as training labels (Gupta et al. 2023; Kosiński et al. 2025).

### Prediction, benchmarking, and cross-system transfer

Eukaryotic PAS prediction targets cleavage and polyadenylation sites, not RNAPII release. Deep models learn positional combinations of PAS hexamers, auxiliary elements, and local structure; in a PolyA-GLM comparison, direct human-to-mouse performance varied across models and datasets, with AUCs of approximately 0.66–0.75 (Arefeen et al. 2019; Stroup et al. 2025; Saha et al. 2026). Interpretation depends on species, tissue, negative-set construction, window definitions, and the sequencing protocols used to generate the labels. Sequence-based PAS models predict cleavage/polyadenylation-related outcomes and do not necessarily capture chromatin-coupled RNAPII termination without information on elongation state, regulatory factors, and chromatin (Eaton and West 2020; Rodríguez-Molina et al. 2023) (Fig. 8b). PolyA_DB 3, PolyASite 2.0, and PlantAPAdb provide cleavage/poly(A)-site labels for training and benchmarking but do not directly label downstream termination windows (Wang et al. 2018; Herrmann et al. 2020; Zhu et al. 2020).

PAS prediction and APA-detection tools can disagree across precision, sensitivity, and differential-APA tasks because read depth, 3′ bias, annotation, gene architecture, and event definitions shape benchmark labels (Tian et al. 2025). PolyAseqTrap addresses protocol heterogeneity and internal priming, but novel sites still require validation (Ye et al. 2026). An intrinsic-terminator classifier is not a Rho predictor, and a poly(A)-site model is not an RNAPII-release model; evaluation therefore requires the measured outcome, host, assay, construct, and condition to be reported and tested experimentally (Fig. 8d, e).

### Generative design, synthetic biology, and biomanufacturing applications

A recent *E. coli* study coupled an SVR terminator-strength predictor with a WGAN-GP generator for intrinsic terminator design (Li et al. 2025). Thirteen of 18 constructed candidates had termination efficiencies above 0.90 in an *E. coli* MG1655 dual-fluorescent reporter assay, providing proof of principle within a small, predictor-filtered, *E. coli*-specific set.

Terminator design applies these findings to expression-boundary control. Mechanistic features explain canonical intrinsic terminator strength but fail on extended, weak, or context-confounded cases, whereas FlowSeq and related assays expand sequence-function coverage (Cambray et al. 2013; Zhai et al. 2022). Cross-host minimal designs, metagenomic screens, and *Halomonas* chassis engineering test terminators as biological parts for expression control and metabolic outputs (Amarelle et al. 2019; Cui et al. 2021; Xu et al. 2022). Their cross-host portability and biomanufacturing utility remain properties to measure in the intended host and pathway context (Fig. 8c).

Massively parallel direct RNA sequencing characterizes engineered transcript isoforms and readthrough or termination outputs, supporting iterative testing in the intended host and assay (Tarnowski and Gorochowski 2022) (Figs. 7b and 8d). Because such assays often use engineered transcription contexts, native RNAP performance remains an empirical question.

Computational design must test whether candidates retain the predicted outcome in the intended host and assay. Hard negatives, failed transfers, and revised labels identify a model’s validated domain and improve the next training dataset (Fig. 8e). Additional comparisons of machine-learning resources, training or reference labels, data sources, validation strategies, strengths, and limitations are summarized in Table S5.

## Technical bottlenecks, future directions, and conclusions

The central technical bottleneck is not a shortage of candidate motifs, mapping technologies, or computational models, but the comparison of results generated by different measurements. The field must determine which observations can be compared directly, which labels correspond to those observations, and whether models or engineered parts remain valid across defined hosts and assays.

### Measurement, dataset, and validation bottlenecks

A recurring problem across Sects. 3, 4, 5, 6 and 7 is that predicted motifs, mapped RNA ends, cleavage sites, readthrough, and polymerase release are different measurements (Fig. 1b; Table 2). Collapsing them into one positive training class can make differences in labels appear as biological disagreement. This problem is especially acute for Rho-dependent and RNAPII systems, where release occurs across a region rather than at a reliably inferred single coordinate.

Term-seq, Rend-seq, TERMITe, PIPETS, long-read and direct RNA approaches, 3′-end atlases, and FLEP-seq resolve complementary RNA ends, isoforms, cleavage/APA sites, and termination windows, but remain affected by processing, stability, truncation, coverage, and endpoint choice (Dar et al. 2016b; Wang et al. 2018; Lalanne et al. 2018; Herrmann et al. 2020; Roth et al. 2020; Mo et al. 2021; Furumo and Meyer 2024; Kosiński et al. 2025). Total RNA-seq, reporter assays, biochemical reconstitution, and direct-release assays add distinct evidence; confidence is strongest when their endpoints converge on the same mechanism in a defined context (Fig. 1b; Table 2).

Auditable records should link signal feature, assay and endpoint, perturbation, condition, inferred mechanism, and host or construct context. Labels should preserve those measurements rather than collapse them into a single “validated terminator” category; species, cell type, genomic context, and assay conditions shape accessibility, factor availability, RNA stability, and measured efficiency.

Robust benchmarking avoids placing homologous or near-duplicate loci in different data partitions and uses motif-matched, expressed, or experimentally tested negatives rather than arbitrary genomic windows. Class prevalence, calibration, data splitting, and external-dataset performance then reveal whether a score reflects biological discrimination or dataset structure. For cross-species or cross-host claims, phylogenetically separated tests or independent host assays are more informative than random internal splits.

Claim-matched validation follows the measurement underlying the intended claim. Independent RNA-end data establish endpoint reproducibility; factor perturbations connect an output to candidate machinery but can retain indirect effects; reporter assays establish function only in the tested construct, host, and condition. Mechanistic claims therefore require convergent evidence from complementary measurements, whereas engineering claims require tests in the intended chassis and pathway. Failed transfers, weak candidates, discordant assays, and difficult negatives identify where a model or terminator does not generalize and improve the next dataset (Fig. 8e).

### Future experimental and machine-learning-guided directions

The central biological question is how particular cis-acting elements, trans-acting factors, and elongation states jointly commit RNAP to release. Progress will depend on studies that pair cis-element perturbations with measurements of RNA-end formation, processing or cleavage, nascent transcription or readthrough, factor dependence, and the relevant translation or chromatin context. Such experiments can distinguish signal features that alter RNA maturation from those that change polymerase progression and identify conditions in which the two processes are causally coupled. Experiments that directly monitor polymerase release and biochemical reconstitution will remain lower throughput, but strategically selected examples can anchor genome-scale reference data and separate mechanistic subclasses that appear similar in RNA-end data.

Machine-learning-guided terminator discovery can combine sequence determinants, RNA structure, accessibility, feature order and spacing, trans-acting factor availability, host identity, and biological condition (Fig. 8b). A model that predicts a cleavage site, readthrough phenotype, reporter efficiency, or termination window requires training or reference data for that specific measurement. Interpretable and multimodal models are useful when they identify informative feature combinations, expose uncertainty, and nominate candidates for experiments appropriate to the predicted measurement.

Generative and optimization models extend this framework from discovery to signal engineering. Their biological value depends on whether designed candidates balance termination strength with host compatibility, neighboring transcription, transcript-isoform consequences, and potential off-target effects. Synthetic libraries, cross-host minimal designs, metagenomic screens, and chassis-specific engineering provide test beds for expression-boundary insulation, reduced transcriptional interference, stable pathway output, and biomanufacturing control (Hudson and Wieden 2019; Amarelle et al. 2019; Cui et al. 2021; Xu et al. 2022). Cross-host portability remains an empirical outcome that requires direct evaluation in the intended genetic circuit, pathway, and production condition.

An iterative design-build-test-learn cycle provides a practical way to test whether a model transfers beyond its training data. Figure 8 organizes observable definition, candidate generation, intended application, matched validation, and dataset curation as a claim-matched workflow. Training datasets can retain experimentally supported positives, weak effects, discordant assays, hard negatives, and failed host transfers so that models prioritize uncertain, diverse, or high-value candidates for testing. This active-learning strategy is a proposed direction, not an established terminator-design standard. Its value depends on whether feedback assays match the intended claim and whether host burden, transcript-isoform control, pathway stability, and production performance are measured in the intended system (Tarnowski and Gorochowski 2022; Li et al. 2025). Progress should be judged by the expansion of a model’s tested host, assay, construct, and condition range, with benchmark performance linked to experimental transfer.

### Conclusions

Across the three domains, termination signals cannot be reduced to interchangeable discrete sequence motifs. Intrinsic hairpin/U-tract modules, Rho-centered RNA-accessibility zones, archaeal U-rich and cleavage-linked routes, and RNAPII PAS-centered windows organize cis-acting signal information at different spatial scales and through different molecular machinery. Treating these systems as operational grammars makes their elements, combinatorial rules, molecular readers, conditional gates, kinetic constraints, and outcomes directly comparable without implying one shared mechanism. These architectures solve shared kinetic and biochemical constraints within distinct cellular organizations; functional similarity and selected component homologies do not imply a linear transition among complete termination pathways. Their shared functional requirement is to create a context in which the elongation complex becomes susceptible to termination, whereas a mapped RNA end, cleavage site, readthrough signal, or release position reports a different stage of that process. Cross-domain synthesis therefore requires claims to remain matched to the observable and biological context that support them.

Future work should determine which signal features causally alter polymerase release rather than RNA maturation, integrate mapping, perturbation, reporter, and direct-release assays across systems, and test whether models trained on endpoint-matched labels retain their expected behavior across hosts and genetic contexts. Resolving these questions will connect mechanism-matched evidence to more reliable prediction and terminator engineering for expression boundaries, transcript outputs, genetic circuits, and biomanufacturing.

## Supplementary Information

Supplementary Material 1.

## Data Availability

This is a review article; no new experimental data were generated. Any supplementary tables accompanying this manuscript are provided as Supporting Information.
