# Supplementary material for: Cross-domain signal grammars of transcription termination
Source: Adv Biotechnol (Singap). 2026 Oct 16;4(4):42. doi: 10.1007/s44307-026-00134-7 (PMC13675443; doi:10.1007/s44307-026-00134-7)
Supplement: Supplementary file 1 [file 44307_2026_134_MOESM1_ESM.docx]

# Supplementary Information

Tables S1–S4 provide additional examples of bacterial intrinsic and Rho-dependent termination, archaeal termination, and eukaryotic nuclear termination discussed in the main text; Table S5 summarizes computational resources, task labels, and validation contexts. Each table retains the signal or measurement, key finding, and scope or limitation needed to interpret the cited study.

## Table S1. Bacterial intrinsic terminators

Examples of bacterial intrinsic terminators, organized by reference and system, signal feature and evidence, key finding, and scope or limitation. The table compares terminator-strength measurements, transcript-end mapping, candidate annotation, cross-host portability, and synthetic-part performance.

| **Reference and system** | **Signal and evidence** | **Key finding** | **Context and limitation** |
| --- | --- | --- | --- |
| Chen et al. (2013) \| bacterial intrinsic termination | Hairpin/U-tract architecture and sequence diversity; 582-member *E. coli* terminator library. | Identified strong terminators with limited shared sequence for recombination-aware circuit design. | *E. coli* synthetic context; cross-species transfer untested. |
| Cambray et al. (2013) \| bacterial intrinsic prediction/resource | GC-rich hairpin/U-tract features; 61-terminator *E. coli* dual-fluorescent assay. | Four features predicted canonical efficiency (r = 0.90; cross-validated r = 0.85; n = 31). | Weak or extended terminators were poorly fit. |
| Mondal et al. (2016) \| bacterial intrinsic termination | Interrupted U-tracts and weak hairpins; RNA-seq plus in vitro/in vivo assays in *B. subtilis*. | NusA promotes termination by supporting hairpin folding and pausing at weak terminators. | Effects are not uniform across terminators. |
| Dar et al. (2016b) \| bacterial intrinsic termination | Conditional RNA structures and premature RNA ends; Term-seq under regulatory conditions. | Ribosome stalling and metabolite-responsive leaders regulate antibiotic-resistance readthrough. | Condition-dependent; broader attenuation mechanisms require validation. |
| Lalanne et al. (2018) \| bacterial intrinsic termination | RNA-end and hairpin/U-tract features; Rend-seq with structural filters and deletion mutants. | Combined end mapping and structure filters identify high-confidence intrinsic terminators. | Thresholds vary by organism; Rho-dependent termination can confound calls. |
| Yan et al. (2018) \| bacterial intrinsic termination | Transcript boundaries and readthrough; SMRT-Cappable-seq full-length primary transcripts. | Readthrough changes operon variants and extends transcript structures. | May miss processed RNAs; condition-specific validation is required. |
| Ju et al. (2019) \| bacterial intrinsic termination | Paired 5′/3′ transcript ends; SEnd-seq full-length profiling. | Revealed widespread bidirectional termination and convergent-boundary effects. | Length/circularization bias and cross-species generality remain uncertain. |
| Bellecourt et al. (2019) \| bacterial intrinsic termination | Hairpin/U-tract and RNAP clamp/trigger-loop perturbations; in vitro kinetics. | Clamp movement aids DNA dissociation but is not essential for RNA release. | In vitro mechanism may not capture cellular context. |
| Hudson and Wieden (2019) \| bacterial intrinsic termination | GC-rich hairpins, extended poly(U) and upstream poly(A) tracts; high-throughput Term-seq of a sequence-diverse synthetic library. | Stronger hairpin pairing and an extended perfect poly(U) tract were associated with higher termination efficiency. | Primarily a synthetic library/resource workflow; mechanistic conclusions should be framed as feature associations. |
| Johnson et al. (2020) \| bacterial intrinsic termination | Stop-codon-to-hairpin distance; comparative genomic and phylogenetic analysis. | Short-distance signatures suggest uncoupled transcription-translation in some lineages. | Computational, lineage-dependent inference rather than direct proof. |
| Gale et al. (2021) \| bacterial intrinsic termination | Hairpin/U-tract architecture; cyanobacterial reporter and sequence-structure tests. | Efficiency varied among cyanobacterial terminators, supporting empirical selection. | Generality outside cyanobacteria is uncertain. |
| Mandell et al. (2021) \| bacterial intrinsic termination | Hairpin/U-tract quality and NusA/NusG perturbations; Term-seq in *B. subtilis*. | NusG cooperates with NusA and promotes pausing at suboptimal terminators. | *B. subtilis*-specific evidence. |
| Adams et al. (2021) \| engineered transcription context | 5′ UTR and ORF-internal RNA ends; RNA-seq and 3′-end mapping in *E. coli*. | Premature termination or processing generates many short RNAs, some with regulatory potential. | Functional roles require targeted tests. |
| Zhai et al. (2022) \| bacterial intrinsic termination | Hairpin/U-tract and thermodynamic features; FlowSeq strength measurements. | Sequence and thermodynamic descriptors distinguish terminator strength. | Synthetic assay context may not capture native diversity. |
| Petroni et al. (2023) \| bacterial intrinsic termination | Upstream/internal RNA ends; Term-seq/RNA-seq in *Borrelia burgdorferi*. | Premature termination or processing generates diverse truncated RNAs and responds to spermidine. | Pathogen-specific atlas; broader effects require validation. |
| Gupta et al. (2023) \| bacterial intrinsic prediction/resource | Predicted single and clustered hairpins; INTERPIN genome database. | Catalogues ~25 million hairpins across 12,745 bacterial genomes; 58% are cluster hairpins. | Computational, chromosome-focused predictions require experimental confirmation. |
| You et al. (2023) \| bacterial intrinsic termination | Pause motif, hairpin, and RNA-DNA hybrid changes; cryo-EM plus biochemical interpretation. | Hairpin completion can promote release through DNA rewinding and hybrid disruption. | Structures support multiple routes, not a single in vivo trajectory. |
| Bar et al. (2023) \| bacterial intrinsic termination | RNAtag-seq read-start patterns; TRS computational terminus calling. | Infers transcript termini without terminus-specific libraries. | Calls depend on protocol-specific read patterns. |
| Cui et al. (2021) \| cross-host bacterial engineering | Minimal hairpin/U-tract designs; dual-fluorescent tests in four bacterial hosts. | Designed terminators performed efficiently across the tested hosts. | Four strains do not establish general portability. |
| Amarelle et al. (2019) \| cross-host bacterial engineering | Metagenomic candidates; terminator-trap and reporter tests across Proteobacteria. | Several candidates were active in multiple Proteobacterial hosts. | Mechanisms and broader host transfer remain unresolved. |
| Xu et al. (2022) \| cross-host bacterial engineering | Hairpin/poly-U designs; reporter and PHB-production tests in *Halomonas*. | Terminator strength was linked to phaC expression and PHB output. | Single-host pathway context limits transfer. |

## Table S2. Bacterial Rho-dependent termination

Examples of Rho-dependent termination, organized by reference and system, signal feature and evidence, key finding, and scope or limitation. The table compares *rut*-like features, factor perturbations, readthrough measurements, RNA-end maps, and termination-zone analyses.

| **Reference and system** | **Signal and evidence** | **Key finding** | **Context and limitation** |
| --- | --- | --- | --- |
| Peters et al. (2009) \| bacterial Rho-dependent termination | Rho perturbation and transcript/RNAP mapping in *E. coli*. | Rho broadly suppresses intragenic and stable-RNA transcription. | Genome-wide regions are termination zones, not precise *rut* sites. |
| Peters et al. (2012) \| bacterial Rho-dependent termination | Rho/NusG perturbation and antisense-transcript mapping in *E. coli*. | Rho and NusG suppress pervasive antisense transcription. | Readthrough phenotypes do not fix *rut* or release positions. |
| Elgamal et al. (2016) \| bacterial Rho-dependent termination | EF-P-dependent translation coupling; profiling and reporter tests. | Relief of ribosome stalling reduces premature Rho termination. | Covers EF-P/polyproline coupling, not all *rut* logic. |
| Ray-Soni et al. (2016) \| bacterial Rho-dependent termination | Review synthesis of intrinsic and Rho-dependent termination. | Factors modulate pausing and termination efficiency across bacterial systems. | Background synthesis rather than primary evidence. |
| Qayyum et al. (2016) \| bacterial Rho-dependent termination | Overlapping NusA/Rho RNA-binding regions; mutants and competition assays in *E. coli*. | NusA can antagonize Rho by competing for RNA access. | *E. coli* evidence; detailed kinetics need targeted tests. |
| Kriner and Groisman (2017) \| bacterial Rho-dependent termination | *rut* accessibility and leader RNA structure; mutagenesis and termination assays. | Structure regulates Rho loading and downstream termination-region function. | Specific leader context limits generalization. |
| Botella et al. (2017) \| bacterial Rho-dependent termination | Rho depletion and transcriptome analysis in *Mycobacterium tuberculosis*. | Rho suppresses pervasive transcription and is required for growth. | Does not map individual *rut* sites; effects are species specific. |
| Bastet et al. (2017) \| bacterial Rho-dependent termination | thiM C-rich *rut* and downstream stem-loop; in vitro and reporter assays. | Translation control and Rho termination are coupled in thiM regulation. | Single riboswitch context. |
| Dar and Sorek (2018) \| bacterial Rho-dependent termination | Term-seq/RNA-seq with Rho inhibition and exonuclease-deficient contexts. | Protective stem-loops separate steady-state RNA ends from release sites. | Mapped ends may reflect processing after termination. |
| Roberts (2019) \| bacterial Rho-dependent termination | Review synthesis of bacterial termination mechanisms. | Summarizes intrinsic signal elements and RNAP conformational mechanisms. | Background synthesis, not primary quantitative evidence. |
| Di Salvo et al. (2019) \| bacterial Rho prediction | 78-nt C-rich/G-poor *rut* candidates plus downstream pause features; RhoTermPredict. | Reported F1 values were ~0.7 in *E. coli* and ~0.6 in *B. subtilis*. | RNA-seq-gradient validation is condition dependent and does not locate release. |
| Song et al. (2022) \| bacterial Rho-dependent termination | Single-molecule/in vitro analysis of catch-up and stand-by routes. | Rho termination can follow multiple routes set by loading and pause timing. | Cellular route usage remains unresolved. |
| Chhakchhuak and Sen (2022) \| bacterial Rho-dependent termination | RT-qPCR of 72 *E. coli* terminators with Rho/NusG perturbations. | Weak/moderate terminators track C>G features and NusG dependence more than strong sites. | Semi-quantitative, selected *E. coli* set. |
| Wang et al. (2023) \| bacterial Rho-dependent termination | sysRNA stabilization with 3′ RACE and primer extension. | Mapped one Rho-dependent region to 21 bp in vivo. | Target-specific stabilization may introduce RNase III-related artifacts. |
| D’Halluin et al. (2023) \| bacterial Rho-dependent termination | Translated uORFs and Rho perturbation in *M. tuberculosis* leaders. | uORF translation associates with altered Rho access and premature termination. | Causality remains locus- and species-specific. |
| Ahmad et al. (2023) \| bacterial Rho-dependent termination | Rho perturbation and termination profiling in *M. tuberculosis*. | Rho has a major role in the high-GC mycobacterial transcriptome. | Dominance claims require species-specific comparative quantification. |
| Molodtsov et al. (2023) \| bacterial Rho-dependent termination | Cryo-EM of *E. coli* Rho-NusG elongation complexes. | NusG bridges a pre-termination complex poised for Rho translocation. | Structural state should not be generalized to all Rho events. |
| Song et al. (2023) \| bacterial Rho-dependent termination | Single-molecule pause and route measurements in terminator/riboswitch contexts. | Pause extension preferentially favors stand-by over catch-up termination. | Limited in vitro systems leave in vivo route usage unresolved. |
| Delaleau et al. (2025) \| bacterial Rho-dependent termination | H-SELEX mapping of *Bacillus* and *E. coli* Rho utilization sites. | Rho substrate preferences differ with RNA structure and accessibility. | In vitro *rut* sites may not be engaged in vivo. |

## Table S3. Archaeal termination-related signals

Examples of archaeal termination-related studies, organized by reference and system, signal feature and evidence, key finding, and scope or limitation. The table covers U-rich tracts, cleavage-linked end formation, transcript-end observations, and aCPSF1/FttA-family factor-associated examples. Its selective entry set reflects the smaller number of studies that directly resolve archaeal termination mechanisms and does not represent all archaeal transcription research.

| **Reference and system** | **Signal and evidence** | **Key finding** | **Context and limitation** |
| --- | --- | --- | --- |
| Santangelo and Reeve (2006) \| archaeal termination | Oligo-T/U tracts and optional hairpins; in vitro archaeal RNAP assays. | Archaeal RNAP can terminate at U/T-rich tracts without an upstream hairpin. | Foundational in vitro evidence; in vivo relevance remains limited. |
| Dar et al. (2016a) \| archaeal termination | U-rich tracts and 3′-end mapping in two archaeal species. | >30% of genes generated alternative 3′ UTR isoforms through multiple terminators. | Two-species comparison; broader prevalence needs validation. |
| Walker et al. (2017) \| archaeal termination | Eta-dependent disruption of stalled elongation complexes; purified *T. kodakarensis* system. | Eta releases stalled/arrested complexes and affects growth and DNA-damage phenotypes. | Kinetic and structural mechanism need further definition. |
| Maier and Marchfelder (2019) \| archaeal termination | Review of archaeal transcription and U/T-rich termination context. | Termination protects compact archaeal transcript architecture. | Background synthesis, not primary evidence. |
| Wenck and Santangelo (2020) \| archaeal termination | Review of archaeal RNAP and post-initiation regulation. | Termination coordinates dense gene expression in a eukaryote-like RNAP system. | Background synthesis, not quantitative evidence. |
| Yue et al. (2020) \| archaeal termination | U-rich signals and aCPSF1 depletion/replacement in *M. maripaludis*. | aCPSF1 depletion causes genome-wide termination defects; distant orthologs can replace it. | Euryarchaeal model; U-rich recognition mechanism needs structural evidence. |
| Sanders et al. (2020) \| archaeal termination | FttA/CPSF73 cleavage of stalled elongation complexes; in vitro assays. | FttA can terminate transcription by cleaving nascent RNA. | Residual-fragment fate and complex disassembly remain unresolved. |
| Berkemer et al. (2020) \| archaeal termination | Genome-wide 3′ termini with reporter tests in *Haloferax volcanii*. | Intergenic T-stretches and coding-region AGATC motifs associate with termini. | One halophile; RNA ends do not resolve mechanism. |
| Li et al. (2021) \| archaeal termination | U-tract recognition by aCPSF1; 3′-end mapping and binding/function assays. | aCPSF1 binds U-tracts and supports termination at weak intrinsic signals. | Quantitative binding and domain effects need cautious wording. |
| Pilotto and Werner (2022) \| archaeal termination | Review of viral/host RNAP inhibition in archaea. | RIP and TFS4 can shut down transcription without canonical terminator motifs. | Contextual shutdown mechanisms, not ordinary terminator signals. |
| You et al. (2024) \| archaeal termination | Cryo-EM and cleavage mapping of FttA/aCPSF1 complexes. | Spt5 bridges FttA to the elongation complex and stimulates cleavage-linked termination. | In vitro system; similarity to Rho is functional, not identity. |

## Table S4. Eukaryotic nuclear transcription-termination pathways and 3′-end processing

Representative studies of PAS-centered RNAPII termination, APA, readthrough, Pol I and Pol III termination, and transcript-class-specific RNAPII pathways, organized by system, signal or evidence, main finding, and interpretation limit.

| **Reference and system** | **Signal and evidence** | **Key finding** | **Context and limitation** |
| --- | --- | --- | --- |
| Schönemann et al. (2014) \| eukaryotic RNAPII 3′ termination | AAUAAA recognition by reconstituted human CPSF. | WDR33-containing CPSF is central to PAS recognition. | Reconstitution does not capture full cellular regulation. |
| Vilborg et al. (2015) \| eukaryotic RNAPII 3′ termination | Stress-induced downstream-of-gene transcription in human cells. | Stress can induce widespread DoG/readthrough transcripts. | Condition-specific; causal factor changes require follow-up. |
| Tian and Manley (2017) \| eukaryotic RNAPII 3′ termination | Review of APA and PAS choice. | APA diversifies isoforms, often through 3′ UTR changes. | Conceptual synthesis, not primary quantitative evidence. |
| Neve et al. (2017) \| eukaryotic RNAPII 3′ termination | Review of PAS elements and CPA machinery. | Cleavage/polyadenylation couples 3′-end formation to RNAPII termination. | Conceptual synthesis rather than primary structural evidence. |
| Baejen et al. (2017) \| eukaryotic RNAPII 3′ termination | Yeast genome-wide analysis of Spt5 and Rat1/Xrn2-dependent termination. | Spt5 contributes to the transition from elongation to termination. | Yeast occupancy metrics do not directly define human PAS-associated sequence organization. |
| Clerici et al. (2017) \| eukaryotic RNAPII 3′ termination | Structural/biochemical assembly of human CPA around AAUAAA. | Clarified CPSF organization during PAS recognition. | Cellular regulation requires endogenous-locus evidence. |
| Sun et al. (2018) \| eukaryotic RNAPII 3′ termination | Cryo-EM and biochemical PAS recognition by CPSF. | WDR33 and CPSF30 directly recognize AAUAAA. | Structural recognition does not alone establish cellular release. |
| Wang et al. (2018) \| eukaryotic RNAPII 3′ termination | PolyA_DB 3 atlas of 3′-sequencing cleavage and poly(A) sites. | Provides curated PAS, 3′ UTR, APA, and sample metadata. | Atlas labels are not direct mechanistic evidence. |
| Clerici et al. (2018) \| eukaryotic RNAPII 3′ termination | Structural analysis of AAUAAA recognition by human CPSF. | Explains canonical PAS specificity. | Noncanonical PAS effects require sequence-function evidence. |
| Eaton and West (2018) \| eukaryotic RNAPII 3′ termination | Review of Xrn2/Rat1 torpedo termination. | Cleavage exposes downstream RNA to exonuclease-driven release. | Mechanistic claims need primary-study support. |
| Mayr (2019) \| eukaryotic RNAPII 3′ termination | Review of APA and 3′ UTR functions. | APA changes post-transcriptional regulation beyond cleavage choice. | Not primary evidence for termination mechanism. |
| Kumar et al. (2019) \| eukaryotic RNAPII 3′ termination | Review of PAS elements and CPA complexes. | CPA integrates PAS and flanking-element recognition for 3′-end processing. | Primary structural claims need original studies. |
| Zhu et al. (2020) \| eukaryotic RNAPII 3′ termination | PlantAPAdb atlas of plant PAS/APA sites. | Provides poly(A)-site, 3′ UTR, and tissue/species annotations. | Resource labels are not direct mechanism evidence. |
| Herrmann et al. (2020) \| eukaryotic RNAPII 3′ termination | PolyASite 2.0 multi-species poly(A)-site atlas. | Consolidates 3′-sequencing site and sample annotations. | Does not establish individual-site function. |
| Vainberg Slutskin et al. (2019) \| eukaryotic RNAPII 3′ termination | Massively parallel PAS reporter assay. | Local sequence context tunes polyadenylation-mediated expression. | Reporter context does not capture chromatin-coupled termination. |
| Yu et al. (2019) \| eukaryotic RNAPII 3′ termination | FY-mutant transcriptome analysis in plants. | FY contributes to plant APA and PAS choice. | Plant-specific; mammalian extrapolation is limited. |
| Sun et al. (2020) \| eukaryotic RNAPII 3′ termination | Review of canonical CPA structure and function. | Summarizes PAS recognition and cleavage mechanisms. | Use primary studies for specific mechanisms. |
| Eaton and West (2020) \| eukaryotic RNAPII 3′ termination | Review of RNAPII torpedo and allosteric termination models. | Termination is coupled to processing and can involve multiple mechanisms. | Conceptual framing, not primary evidence. |
| Yang et al. (2021) \| eukaryotic RNAPII 3′ termination | Review of plant co-transcriptional 3′-end processing. | Plant PAS processing is coordinated with RNAPII. | Broad mechanism and LLPS claims need primary support. |
| Rosa-Mercado and Steitz (2022) \| eukaryotic RNAPII 3′ termination | Review of stress-induced DoG/readthrough transcription. | Stress can induce downstream-of-gene transcripts. | Proposed DoG functions need perturbation evidence. |
| Han et al. (2023) \| eukaryotic RNAPII 3′ termination | T-rich DNA elements and cleavage-independent RNAPII termination. | T-rich sequences can direct termination outside canonical CPA. | Generality relative to PAS/Xrn2 requires validation. |
| Rodríguez-Molina et al. (2023) \| eukaryotic RNAPII 3′ termination | Review of PAS-dependent and factor-coupled RNAPII termination. | Protein-coding-gene termination is coordinated with 3′-end processing. | Conceptual synthesis; use primary mechanism studies. |
| Davidson et al. (2024) \| eukaryotic RNAPII 3′ termination | Genomic and mechanistic tests of DNA T-rich termination elements. | Mammalian RNAPII can terminate at DNA-encoded T-rich signals. | Scope and contribution relative to CPA/Xrn2 remain to be defined. |
| Caldas et al. (2024) \| eukaryotic RNAPII 3′ termination | Healthy-human-tissue atlas of downstream readthrough RNA. | Readthrough is prevalent and associates with tissue and genomic features. | Observational atlas does not establish causality. |
| Nielsen et al. (2025) \| eukaryotic Pol I termination | Defined-scaffold release assays; with/without upstream hairpin | Conserved pre-rRNA hairpin required for efficient Pol I release | In vitro scaffold; cross-lineage conservation inferred |
| Petfalski et al. (2025) \| eukaryotic Pol I termination | Genome-wide and biochemical analyses of Rnt1, Rat1, Rpa12 | No single obligatory Pol I route; premature termination modeled | Budding yeast; modeled estimates, not direct event counts |
| Xie et al. (2022) \| eukaryotic Pol III termination | T tracts, hairpins, Sen1 fail-safe; CRAC and in vitro assays | T tracts not always sufficient; hairpins/Sen1 enhance weak terminators | Budding yeast; generalizability to other Pol III systems untested |
| Girbig et al. (2022) \| eukaryotic Pol III termination | Cryo-EM of Pol III pre-termination complexes on poly(dT) | C128 recognition of poly(dT) stabilizes paused pre-termination state | Structural capture; final release steps modeled |
| Xiong et al. (2024) \| specialized RNAPII NNS/Sen1 | Single-molecule reconstitution of Sen1 on stalled Pol II | Sen1 releases RNA in ATP-coupled step; translocation ~80 nt/s | Purified engineered system; not a cellular release census |
| Baillat et al. (2005) \| specialized RNAPII Integrator | Integrator-CTD association at U1/U2 snRNA genes | Integrator required for U1/U2 precursor 3′-end processing | Processing endpoints; no direct RNAPII release measurement |
| Sabath et al. (2024) \| specialized RNAPII Integrator | Transcription-factor contacts recruit Integrator to promoters | Gene-specific targeting; required for glucose-starvation responses | Recruitment endpoints; does not count release events |
| Ielasi et al. (2022) \| specialized RNAPII histone transcripts | Overlapping PAS and HDE elements in histone pre-mRNAs | Overlapping elements recruit canonical or histone-specific complexes | Processing-complex choice, not direct polymerase release |

## Table S5. Machine-learning resources, training labels, and design-oriented studies

Comparisons of machine-learning resources, training labels, and design-oriented studies, organized by reference, task and label, approach and data, and key result and caveat. The table distinguishes bacterial terminator prediction, Rho-dependent prediction, PAS/poly(A)-site prediction, 3′-end resource construction, termination-window modeling, and synthetic terminator design.

| **Reference** | **Task and label** | **Approach and data** | **Key result and caveat** |
| --- | --- | --- | --- |
| Chen et al. (2013) | Intrinsic terminator design; library-measured strength. | 582-member *E. coli* natural/synthetic library; hairpin/U-tract architecture and sequence diversity. | Strong terminators with limited shared sequence supported recombination-aware circuit design; Caveat: *E. coli* synthetic context, with cross-species transfer untested. |
| Cambray et al. (2013) | Intrinsic terminator prediction; reporter-measured efficiency. | *E. coli* dual-fluorescent assay of 61 terminators with folding and sequence-function analysis. | Four features predicted canonical efficiency (r = 0.90; cross-validated r = 0.85; n = 31); Caveat: weak or extended terminators were poorly fit. |
| Baejen et al. (2017) | RNAPII termination; PAS-associated downstream release. | Yeast genome-wide analysis of Spt5 and Rat1/Xrn2-dependent termination. | Rat1/Xrn2 contributes downstream of polyadenylation sites; Caveat: yeast occupancy metrics do not directly define human PAS-associated sequence organization. |
| Wang et al. (2018) | PAS/APA resource; cleavage and poly(A)-site labels. | PolyA_DB 3 catalogues 3′-sequencing-derived sites, motifs, 3′ UTRs, and tissue/species metadata. | Supports PAS and APA annotation across datasets; Caveat: an atlas, not direct mechanistic evidence. |
| Dar and Sorek (2018) | Rho-dependent termination; mapped steady-state RNA ends. | Term-seq/RNA-seq with Rho inhibition and exonuclease-deficient contexts in *E. coli*. | Protective stem-loops can separate observed 3′ ends from release sites; Caveat: RNA ends may reflect downstream processing. |
| Feng et al. (2019) | Intrinsic terminator prediction; annotated RNA termini. | iTerm-PseKNC SVM using sequence composition and physicochemical correlations. | Reported 95% cross-validation accuracy; Caveat: small, sequence-only benchmarks do not separate intrinsic and Rho-dependent classes. |
| Arefeen et al. (2019) | PAS prediction; poly(A)-site and tissue-dominance labels. | DeepPASTA CNN/RNN with sequence windows and predicted RNA structure. | Structure and RNN integration improved benchmark prediction; Caveat: human polyadenylation labels do not directly measure RNAPII release. |
| Zhu et al. (2020) | Plant PAS/APA resource; annotated poly(A)-site labels. | PlantAPAdb integrates sites, 3′ UTR isoforms, and tissue/species metadata. | Supports comparative PAS and APA analysis; Caveat: atlas completeness depends on input datasets and annotation. |
| Herrmann et al. (2020) | PAS/APA resource; integrated poly(A)-site labels. | PolyASite 2.0 integrates 3′-end sequencing sites, motifs, and sample metadata. | Supports cross-dataset PAS/APA analysis; Caveat: does not establish the function of individual sites. |
| Hudson and Wieden (2019) | Synthetic terminator design; sequence-function measurements. | High-throughput terminator libraries with Term-seq and structural features. | Hairpin pairing and extended poly(U) tracts associate with higher efficiency; Caveat: associations are not complete mechanism tests. |
| Di Salvo et al. (2019) | Rho-dependent prediction; *rut*-like RNA and pause features. | RhoTermPredict scans 78-nt C-rich/G-poor *rut* candidates plus downstream pause signals. | Separates Rho and intrinsic prediction tasks; Caveat: RNA-seq-gradient validation is condition dependent and does not fix a release coordinate. |
| Roth et al. (2020) | Readthrough detection; downstream-RNA phenotype. | ARTDeco quantifies readthrough, read-in, and DoG RNA from aligned RNA-seq data. | Readthrough alters expression and eQTL interpretation; Caveat: it detects downstream RNA, not primary terminator motifs. |
| Berkemer et al. (2020) | Archaeal RNA-end atlas; mapped 3′ termini. | TEX-treated RNA-seq peak calling with reporter tests in *Haloferax volcanii*. | Intergenic T-stretches and coding-region AGATC motifs associate with termini; Caveat: one halophile and RNA ends do not resolve mechanism. |
| Tarnowski and Gorochowski (2022) | Engineered terminator design; transcript-isoform output. | Massively parallel direct RNA sequencing of transcriptional valves. | Defines sequence-to-isoform design relationships; Caveat: primarily an engineered T7 RNAP context. |
| Zhai et al. (2022) | Intrinsic terminator design; high-throughput strength. | FlowSeq links terminator strength to sequence and thermodynamic features. | Strong hairpin/U-tract architectures support strength prediction; Caveat: synthetic assays do not cover all native contexts. |
| Gupta et al. (2023) | Intrinsic-terminator resource; predicted hairpin candidates. | INTERPIN mines bacterial genomes for single and clustered termination hairpins. | Cluster hairpins comprised 58% of inferred entries; Caveat: computational chromosome-focused predictions require experimental confirmation. |
| Wang et al. (2023) | Rho-dependent region mapping; stabilized RNA ends. | sysRNA coupled to 3′ RACE and primer extension in vivo. | Narrowed one Rho-dependent region to 21 bp; Caveat: target-specific stabilization may introduce RNase III-related artifacts. |
| Bar et al. (2023) | Transcript-terminus calling; RNAtag-seq read-start patterns. | TRS infers RNA termini from existing RNAtag-seq datasets. | Extends terminus analysis without a dedicated assay; Caveat: calls depend on protocol-specific read patterns. |
| Caldas et al. (2024) | Human readthrough atlas; downstream-RNA phenotype. | Genomic analysis of readthrough transcripts in healthy tissues. | Readthrough associates with tissue and genomic features; Caveat: observational data do not establish causality. |
| Stroup et al. (2025) | Polyadenylation-model review; PAS and APA labels. | Review of CNN and CNN/RNN models using PAS motifs and auxiliary elements. | Models predict site class, strength, APA, or variants; Caveat: conclusions depend on the underlying assays and do not cover all transcriptional context. |
| Kosiński et al. (2025) | Bacterial terminator atlas; reproducible 3′ RNA ends. | TERMITe calls replicated Term-seq peaks and annotates hairpin/U-tract features. | Across 13 species, supported terminators varied in sequence and structure; Caveat: stable ends can include processing products. |
| Delaleau et al. (2025) | Rho-site mapping; in vitro *rut* affinity. | H-SELEX compares *Bacillus* and *E. coli* Rho substrate preferences. | Identifies sequence/accessibility preferences; Caveat: in vitro sites need not be used in vivo. |
| Li et al. (2025) | Intrinsic terminator design; reporter-measured efficiency. | SVR strength predictor plus WGAN-GP generator using *E. coli* sequence, thermodynamic, and RNA-structure features. | 13/18 generated candidates exceeded 0.90 efficiency in an *E. coli* reporter; Caveat: small predictor-filtered set with untested cross-host transfer. |
| Cui et al. (2021) | Minimal terminator design; cross-host reporter efficiency. | In silico design with dual-fluorescent tests in four bacterial hosts. | Validated in *B. subtilis*, *C. glutamicum*, and *E. coli*; Caveat: four strains do not establish universal portability. |
| Amarelle et al. (2019) | Metagenomic terminator discovery; cross-host reporter activity. | Terminator-trap screening in *Pseudomonas putida* with Proteobacterial validation. | Active candidates transferred among several Proteobacteria; Caveat: mechanisms and broader host range remain unresolved. |
| Xu et al. (2022) | Chassis-specific terminator design; reporter efficiency and PHB output. | RNA-seq mining and reporter/production tests in *Halomonas bluephagenesis*. | Linked terminator performance to a production phenotype; Caveat: single-host evidence and pathway effects limit transfer. |
| Taheri Ghahfarokhi and Peña-Castillo (2025) | Bacterial terminator prediction; annotated termination-site labels. | CNN ensemble trained on ~41,000 sites from 22 species using 100-nt sequence windows. | Caveat: Performance declines with GC content and does not separate intrinsic from factor-dependent mechanisms. |
| Saha et al. (2026) | PAS prediction; balanced cleavage-site labels. | DNABERT-2, Nucleotide Transformer, and HyenaDNA classify 101-bp poly(A)-site windows. | Tests sequence-feature use across models; Caveat: DNA-only inputs omit RNA structure and factor context. |
| Tian et al. (2025) | PAS/APA benchmarking; tool calls and differential-APA events. | Five PAS predictors and seven APA tools compared across ccRCC RNA-seq datasets. | Tool agreement and precision/sensitivity depend on the use case; Caveat: depth, 3′ bias, and reference coverage limit labels. |
| Ye et al. (2026) | PAS label cleaning; poly(A)-site and internal-priming calls. | Cross-protocol 3′-end pipeline with weighted peak clustering and DeepIP filtering. | Improves cross-protocol comparison; Caveat: intronic and novel calls still require biological validation. |
